# Brain Health After COVID-19, Pneumonia, Myocardial Infarction, or Critical Illness

**DOI:** 10.1001/jamanetworkopen.2023.49659

**Published:** 2023-12-28

**Authors:** Costanza Peinkhofer, Pardis Zarifkar, Rune Haubo B. Christensen, Vardan Nersesjan, Lise Fonsmark, Charlotte Merie, Anne-Mette Lebech, Terese Lea Katzenstein, Lia Evi Bang, Jesper Kjærgaard, Pradeesh Sivapalan, Jens-Ulrik Stæhr Jensen, Michael Eriksen Benros, Daniel Kondziella

**Affiliations:** 1 Department of Neurology, Rigshospitalet, Copenhagen University Hospital, Copenhagen, Denmark; 2 Copenhagen Research Center for Biological and Precision Psychiatry, Mental Health Centre Copenhagen, Copenhagen University Hospital, Copenhagen, Denmark; 3 Department of Intensive Care, Rigshospitalet, Copenhagen University Hospital, Copenhagen, Denmark; 4 Department of Infectious Diseases, Rigshospitalet, Copenhagen University Hospital, Copenhagen, Denmark; 5 Department of Clinical Medicine, University of Copenhagen, Copenhagen, Denmark; 6 Department of Cardiology, Rigshospitalet, Copenhagen University Hospital, Copenhagen, Denmark; 7 Section of Respiratory Medicine, Department of Medicine, Copenhagen University Hospital Herlev and Gentofte, Hellerup, Denmark

## Abstract

**Importance:**

Brain health is most likely compromised after hospitalization for COVID-19; however, long-term prospective investigations with matched control cohorts and face-to-face assessments are lacking.

**Objective:**

To assess whether long-term cognitive, psychiatric, or neurological complications among patients hospitalized for COVID-19 differ from those among patients hospitalized for other medical conditions of similar severity and from healthy controls.

**Design, Setting, and Participants:**

This prospective cohort study with matched controls was conducted at 2 academic hospitals in Copenhagen, Denmark. The case cohort comprised patients with COVID-19 hospitalized between March 1, 2020, and March 31, 2021. Control cohorts consisted of patients hospitalized for pneumonia, myocardial infarction, or non–COVID-19 intensive care–requiring illness between March 1, 2020, and June 30, 2021, and healthy age- and sex-matched individuals. The follow-up period was 18 months; participants were evaluated between November 1, 2021, and February 28, 2023.

**Exposures:**

Hospitalization for COVID-19.

**Main Outcomes and Measures:**

The primary outcome was overall cognition, assessed by the Screen for Cognitive Impairment in Psychiatry (SCIP) and the Montreal Cognitive Assessment (MoCA). Secondary outcomes were executive function, anxiety, depressive symptoms, and neurological deficits.

**Results:**

The study included 345 participants, including 120 patients with COVID-19 (mean [SD] age, 60.8 [14.4] years; 70 men [58.3%]), 125 hospitalized controls (mean [SD] age, 66.0 [12.0] years; 73 men [58.4%]), and 100 healthy controls (mean [SD] age, 62.9 [15.3] years; 46 men [46.0%]). Patients with COVID-19 had worse cognitive status than healthy controls (estimated mean SCIP score, 59.0 [95% CI, 56.9-61.2] vs 68.8 [95% CI, 66.2-71.5]; estimated mean MoCA score, 26.5 [95% CI, 26.0-27.0] vs 28.2 [95% CI, 27.8-28.6]), but not hospitalized controls (mean SCIP score, 61.6 [95% CI, 59.1-64.1]; mean MoCA score, 27.2 [95% CI, 26.8-27.7]). Patients with COVID-19 also performed worse than healthy controls during all other psychiatric and neurological assessments. However, except for executive dysfunction (Trail Making Test Part B; relative mean difference, 1.15 [95% CI, 1.01-1.31]), the brain health of patients with COVID-19 was not more impaired than among hospitalized control patients. These results remained consistent across various sensitivity analyses.

**Conclusions and Relevance:**

This prospective cohort study suggests that post–COVID-19 brain health was impaired but, overall, no more than the brain health of patients from 3 non–COVID-19 cohorts of comparable disease severity. Long-term associations with brain health might not be specific to COVID-19 but associated with overall illness severity and hospitalization. This information is important for putting understandable concerns about brain health after COVID-19 into perspective.

## Key Points

**Question:**

Do patients hospitalized for COVID-19 have long**-**term cognitive, psychiatric, or neurological complications compared with healthy controls and matched patients hospitalized for non–COVID-19 medical conditions of similar severity?

**Findings:**

In this matched cohort study including 345 participants, patients hospitalized for COVID-19 performed worse than healthy controls on cognitive, psychiatric, and neurological tests. However, compared with hospitalized controls matched for age, sex, and severity of disease, the impairment of brain health was similar.

**Meaning:**

This study suggests that the brain health of patients was impaired after severe COVID-19, but no more than the brain health of patients hospitalized for other diseases of similar severity.

## Teaser

This matched cohort study assesses whether long-term cognitive, psychiatric, or neurological complications among patients hospitalized for COVID-19 differ from those among patients hospitalized for other medical conditions of similar severity and from healthy controls.

## Introduction

Impaired brain health after SARS-CoV-2 infection remains common 3 years after the outbreak of COVID-19,^1^ echoing impairments seen in previous virus pandemics.^2,3^ The long-term effects of COVID-19 are associated with more than 200 symptoms, affecting 65 million individuals worldwide.^4,5^ Apart from respiratory symptoms, the most frequent symptoms are related to brain health, including cognitive and mental symptoms.^6,7,8^ However, long-term cognitive and neuropsychiatric sequelae also occur after non–COVID-19 conditions, such as pneumonia,^9^ myocardial infarction,^10^ and other diseases, including those requiring admission to the intensive care unit (ICU).^11^

Available studies on brain health impairment after COVID-19 compared with that after non–COVID-19 diseases are based mostly on electronic health records or surveys, lack adequate controls, and often focus on self-assessed symptoms and not objective clinical investigations.^12,13,14^ Hence, to discern the nature, extent, and trajectories of brain health complications specific to COVID-19, long-term prospective studies are required that involve clinical in-person evaluations of patients with COVID-19 and controls from cohorts appropriately matched for the degree of critical illness and level of hospitalization.^15^

Here, we performed, to our knowledge, the first prospective, face-to-face, posthospital follow-up to assess whether long-term cognitive, psychiatric, or neurological complications among patients hospitalized for COVID-19 differ from those among healthy controls and carefully matched patients hospitalized for (1) pneumonia, (2) myocardial infarction, and (3) non–COVID-19, ICU-requiring conditions.

## Methods

### Study Design and Participants

Participants were evaluated between November 1, 2021, and February 28, 2023 (eFigure 1 in Supplement 1). eMethods 1 and 2 in Supplement 1 include detailed inclusion and exclusion criteria. The study followed the Strengthening the Reporting of Observational Studies in Epidemiology (STROBE) reporting guideline and was reviewed and approved by the Danish Regional Committee on Health Research Ethics in Copenhagen. All participants gave written consent.

#### COVID-19 Case Cohort

We prospectively enrolled all patients hospitalized for COVID-19 at Rigshospitalet, a tertiary care hospital in Copenhagen, Denmark, from March 1, 2020, to March 31, 2021. Patients hospitalized for COVID-19 during the same time period at another Copenhagen hospital (Gentofte Hospital) were also enrolled.

#### Matched Control Cohorts

Controls were matched for age and sex; hospitalized controls were additionally matched for ICU admission. Control patients were hospitalized between March 1, 2020, and June 30, 2021, for non–COVID-19 pneumonia, myocardial infarction, or non–COVID-19, ICU-requiring illness (eTable 1 in Supplement 1) and were admitted to Rigshospitalet or Gentofte Hospital. Exclusion criteria were previous hospitalization for COVID-19 and recent (<3 months) SARS-CoV-2 infections. Healthy controls were 18 years of age or older and without hospitalization in the preceding 2 years.

### Clinical Follow-Up Assessments

Eligible patients were contacted by telephone and email and invited for in-person follow-up a mean (SD) of 19.4 (1.6) months after discharge (6-month follow-up data are available elsewhere^16^). Participants underwent a structured, face-to-face interview by 2 trained physicians supervised by senior neurology (D.K.) and senior psychiatry (M.E.B.) consultants.

#### Cognitive Assessments

Cognition was assessed using the Screen for Cognitive Impairment in Psychiatry (SCIP; lower scores indicate greater cognitive impairment [minimun 0, no maximum, mild impairment <75]) and the Montreal Cognitive Assessment (MoCA; scores <26 are abnormal and indicate cognitive impairment; lower scores indicate greater cognitive impairment [range, 0-30]).^17,18^ Executive functions were tested with Trail Making Tests A and B (higher scores indicate greater executive dysfunction [maximum time, 5 minutes; Trail A deficient, >78 seconds; Trail B deficient, >273 seconds]).^19^

#### Neuropsychiatric Interview

We evaluated depression and anxiety using the Hamilton Anxiety Rating Scale (HAM-A; higher scores indicate greater anxiety [range, 0-56]) and the Hamilton Depression Rating Scale (HAM-D; higher scores indicate greater depression [range, 0-52]). Patients were screened for newly acquired psychiatric disorders using the Mini International Neuropsychiatric Interview (MINI), version 5.0. Electronic health records were screened to confirm new onset of the disorder, according to the *International Statistical Classification of Diseases and Related Health Problems, Tenth Revision*.

#### Neurological Examination

We assessed sensorimotor and cerebellar functions and cranial nerves, including the 4-item Pocket Smell Test “scratch and sniff” for olfaction (range, 0-4; <4, abnormal). Neurological signs were quantified with the Neurological Evaluation Scale (26 items, rated on a scale from 0 to 2 [no impairment, mild impairment, and severe impariment] except snout and suck reflexes rated only 0 and 2 [no impairment and impairment]).

#### Self-Experienced Symptoms and Fatigue After Hospitalization

We collected subjective cognitive and neuropsychiatric symptoms using semistructured interviews (eMethods 3 in Supplement 1). Symptoms were registered if they were of new onset or had worsened after hospitalization and other causes were excluded. For controls with a previous SARS-COV-2 infection, typical symptoms of COVID-19, such as anosmia and dysgeusia, were documented only if associated with hospitalization. The Fatigue Assessment Scale was administered to investigate mental and physical fatigue. Total scores ranged from 10 to 50 (<22 indicating no fatigue; 10 items, scored from 0 to 5 [never to always]).

#### Basic Clinical Data

**Figure 1.**
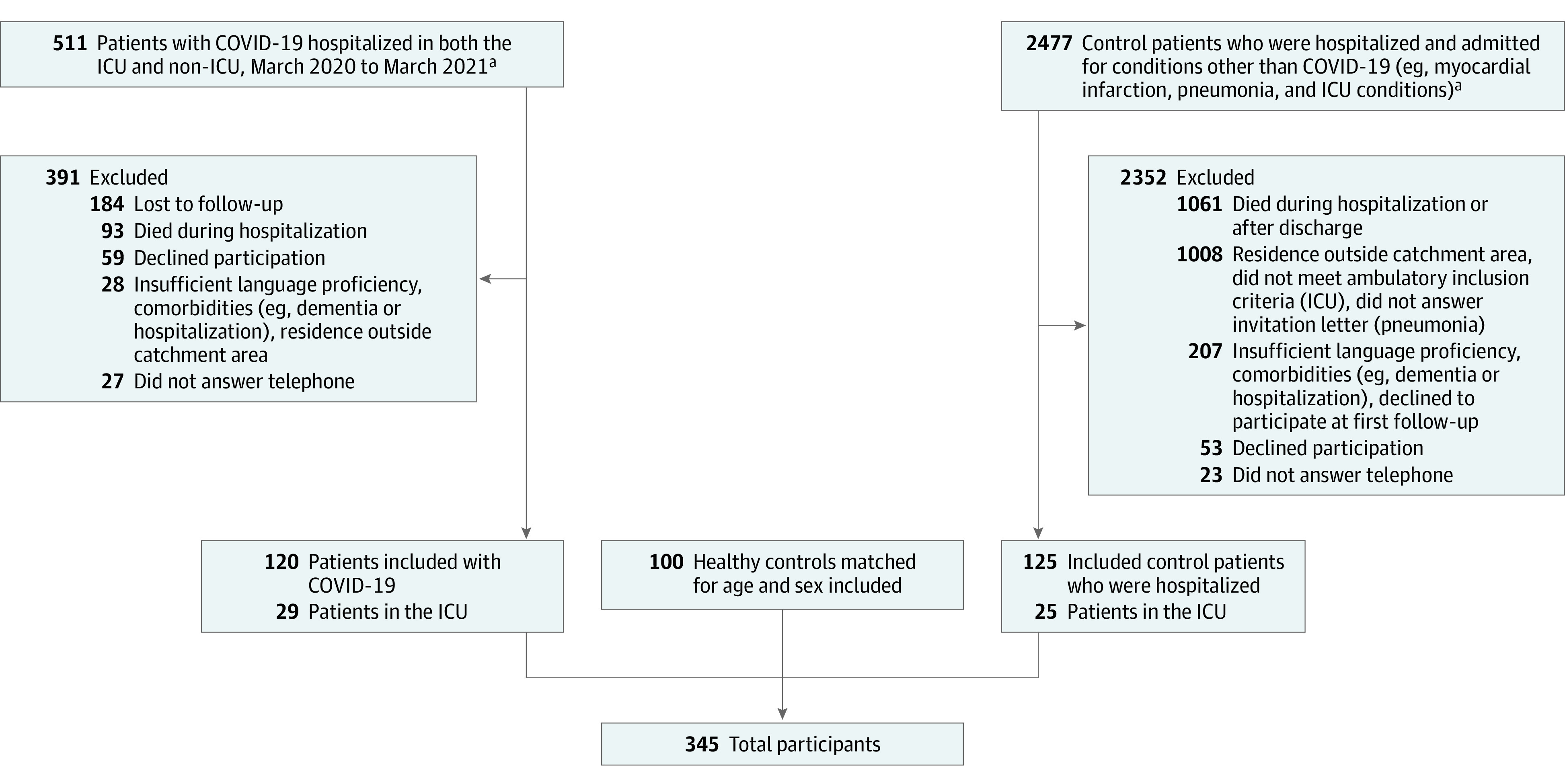
Abbreviated Flowchart of the Recruitment Process for Patients With COVID-19, Patients Hospitalized With Non–COVID-19 Illness, and Healthy Controls See eFigure 1 in Supplement 1 for a detailed flowchart. ^a^A total of 56 of 511 patients with COVID-19 (11.0%) were hospitalized at another hospital during the same time period and considered for follow-up; 3 patients in the intensve care unit (ICU) were included from the group of patients hospitalized with non–COVID-19 illness.

We collected data on age, sex, educational level, comorbidities, smoking status, alcohol consumption, delirium during admission, laboratory test findings, brain imaging, cerebrospinal fluid findings, electroencephalograms, nerve conduction studies, and electromyograms. An ordinal scale was used to record hospital disease severity.^20^ We made 3 attempts to contact eligible individuals by telephone and sent out written invitations before considering them lost to follow-up (Figure 1; eFigure 1 in Supplement 1).

### Primary, Secondary, and Exploratory Outcomes

The primary outcome was overall cognition assessed by the SCIP scores and log-transformed scores (32 – MoCA [all scores are subtracted from 32 and the new value is log-transformed]). Secondary outcomes were log-transformed Trail Making A and B scores, HAM-A and HAM-D scores, and Neurological Evaluation Scale scores. In addition, exploratory outcomes (eMethods 4 in Supplement 1) included changes over time in MoCA score and symptom frequency, neurological examination results, psychiatric diagnoses, number of subjective symptoms, and fatigue.

### Statistical Analysis

Statistical analyis was performed from May 17 to July 18, 2023. First, primary and secondary outcomes were compared among patients hospitalized with COVID-19, other hospitalized individuals, and healthy controls. Second, we compared patients with COVID-19 without ICU admission with control patients with pneumonia and myocardial infarction and healthy controls. Third, patients with COVID-19 requiring ICU admission were compared with controls requiring ICU admission for non–COVID-19 reasons. Comparisons between patients with COVID-19 and hospitalized controls were adjusted for age, sex, ICU admission, total number of admission days, number of days to follow-up, and disease severity. Comparisons with healthy controls were adjusted for sex, age, body mass index (BMI; calculated as weight in kilograms divided by height in meters squared), educational level, alcohol abuse, and smoking. Exploratory outcomes were compared among patients with COVID-19, hospitalized individuals, and healthy controls.

The primary outcome of MoCA score was left skewed and log transformed (32 – MoCA) before analysis. Secondary outcomes were right skewed and transformed with log (outcome) or log (outcome + 1) when necessary. Analyses of continuous outcomes used linear models with estimated mean values back transformed from the log scale and effects interpretable as relative mean changes. Binary outcomes were analyzed in logistic regression models presenting odds ratios (ORs). Ordinal outcomes were analyzed in proportional odds models presenting cumulative ORs. For binary and ordinal outcomes, 95% CIs and *P* values were based on Wald statistics. Effect heterogeneity *P* values were based on likelihood ratio statistics.

Analyses of change for MoCA scores at hospital discharge, 6 months, and 18 months among patients with COVID-19 were performed in linear mixed-effects models with patients as random effects. Sex and age were used as matching variables for adjustment of the models because confounders that remain constant within patients will not alter the results.

Analysis of change for patients with COVID-19 in binary variables at 6-month and 18-month follow-up was performed in logistic regression models with patients as random effects using the 7-point adaptive Gauss-Hermite quadrature for the computations with no confounder adjustment. The 95% CIs for the estimated ORs were based on Wald statistics. *P* values were based on likelihood ratio statistics.

All *P* values were 2-sided, and results were deemed statistically significant at *P* < .05. For exploratory outcomes, *P* values were left unadjusted, and a Bonferroni-adjusted α level of *P* < .001 (0.05/48) was applied to assess significance on a multiplicity-adjusted 5% α level. Missing data were not imputed, and complete-cases analyses were reported. Statistical analyses were conducted in R, version 4.1.2 (R Project for Statistical Computing)^21^ using different packages.^22,23,24^

## Results

We examined 345 participants, including 120 patients with COVID-19 (29 admitted to the ICU; mean [SD] age, 60.8 [14.4] years; 70 men [58.3%] and 50 women [41.7%]), 100 matched healthy controls (mean [SD] age, 62.9 [15.3] years; 46 men [46.0%] and 54 women [54.0%]), and 125 matched control patients (mean [SD] age, 66.0 [12.0] years; 73 men [58.4%] and 52 women [41.6%]) hospitalized during the same time period for pneumonia (n = 50), acute myocardial infarction (n = 50), or an ICU-requiring illness other than COVID-19 (n = 25) (Figure 1; Table 1).

**Table 1.** Baseline Characteristics of Patients With COVID-19, Hospitalized Controls, and Healthy Controls

Characteristic	No. (%)			*P* value^a^	
	Patients with COVID-19 (n = 120)	Hospitalized controls (n = 125)	Healthy controls (n = 100)	Patients with COVID-19 vs hospitalized controls	Patients with COVID-19 vs healthy controls
Demographics					
Age, mean (SD), y	60.8 (14.4)	66.0 (12.0)	62.9 (15.3)	.002	.30
Sex					
Female	50 (41.7)	52 (41.6)	54 (54.0)	.99	.07
Male	70 (58.3)	73 (58.4)	46 (46.0)		
BMI, mean (SD)	29.6 (6.0)	26.2 (4.3)	25.6 (5.2)	<.001	<.001
Smoking					
Current	1 (0.8)	21 (16.8)	9 (9.0)	<.001	.007
Never	59 (49.2)	44 (35.2)	53 (53.0)		
Previous	60 (50.0)	60 (48.0)	38 (38.0)		
Educational level					
Length, mean (SD), y	13.1 (3.1)	13.4 (2.4)	14.8 (2.7)	.45	<.001
Grade					
Primary school	30 (25.0)	17 (13.6)	9 (9.0)	<.001	<.001
Vocational training	29 (24.2)	32 (25.6)	23 (23.0)		
Short cycle^b^	15 (12.5)	28 (22.4)	4 (4.0)		
Medium cycle^b^	27 (22.5)	31 (24.8)	34 (34.0)		
Long cycle^b^	19 (15.8)	17 (13.6)	30 (30.0)		
COVID-19 infection	120 (100.0)	54 (43.2)	54 (54.0)	<.001	<.001
COVID-19 vaccination	114 (95.0)	124 (99.2)	98 (98.0)	.05	.24
Previous medical history					
Any comorbidity	92 (76.7)	104 (83.2)	51 (51.0)	.20	<.001
Hypertension	43 (35.8)	53 (42.4)	28 (28.0)	.29	.22
Hyperlipidemia	30 (25.0)	51 (40.8)	15 (15.0)	.009	.07
Type 2 diabetes	21 (17.5)	10 (8.0)	5 (5.0)	.03	.004
Malignant neoplasm	14 (11.7)	13 (10.4)	9 (9.0)	.75	.52
Autoimmune disorder	12 (10.0)	17 (13.6)	2 (2.0)	.38	.02
Asthma	21 (17.5)	15 (12.0)	3 (3.0)	.22	<.001
Other comorbidities	33 (27.5)	40 (32.0)	9 (9.0)	.44	<.001
Previous psychiatric history					
Any psychiatric comorbidity	41 (34.2)	37 (29.6)	27 (27.0)	.44	.25
Depression	21 (17.5)	21 (16.8)	13 (13.0)	.88	.36
Anxiety	10 (8.3)	9 (7.2)	11 (11.0)	.74	.50
PTSD	2 (1.7)	3 (2.4)	1 (1.0)	.69	.67
Stress	11 (9.2)	13 (10.4)	4 (4.0)	.75	.13
Admission characteristics					
Admission, median (IQR), d	7.0 (4.0-18.0)	5.0 (3.0-14.0)	NA	.12	NA
ICU admission	29 (24.2)	25 (20.0)	NA	.43	NA
Severity					
Not requiring oxygen	26 (21.7)	70 (56.0)	NA	<.001	NA
Requiring oxygen	53 (44.2)	28 (22.4)	NA		
Requiring HFNC or NIV	18 (15.0)	3 (2.40)	NA		NA
Requiring IMV or ECMO	23 (19.2)	24 (19.2)	NA		NA
mRS score at discharge, mean (SD)^c^	1.3 (1.3)	1.8 (1.3)	NA	.005	NA
Laboratory test findings, peak values					
Leukocytes, median (IQR), cells/µL^d^	10 300 (7000-15 600)	13 000 (9500-17 900)	NA	.04	NA
CRP, median (IQR), mg/dL^e^	12.3 (6.5-23.8)	14.9 (4.2-29.2)	NA	.86	NA
Creatinine, median (IQR), mg/dL^f^	1.03 (0.83-1.40)	0.97 (0.88-1.24)	NA	.27	NA
ALT, median (IQR), U/L^g^	75.0 (40.5-124.0)	62.0 (28.2-172.0)	NA	.81	NA
D-dimer, median (IQR), µg/mL^h^	7.7 (2.7-22.9)	8.2 (4.4-20.8)	NA	.46	NA
Creatine kinase, median (IQR), U/L^i^	104.0 (47.0-198.0)	1050.0 (332.0-1980.0)	NA	<.001	NA
LDH, median (IQR), U/L^j^	388.0 (275.5-504.5)	268.5 (200.2-412.8)	NA	.001	NA
Time to follow-up, mean (SD), d	542.1 (167.6)	640.6 (161.9)	NA	<.001	NA

Abbreviations: ALT, alanine aminotransferase; BMI, body mass index (calculated as weight in kilograms divided by height in meters squared); CRP, C-reactive protein; ECMO, extracorporeal membrane oxygenation; HFNC, high-flow nasal cannula; ICU, intensive care unit; IMV, invasive mechanical ventilation; LDH, lactate dehydrogenase; mRS, modified Rankin Scale; NA, not applicable; NIV, noninvasive ventilation; PTSD, posttraumatic stress disorder.

SI conversion factors: To convert leukocytes to cells ×10^9^ per liter, multiply by 0.001; CRP to milligrams per liter, multiply by 10.0; creatinine to micromoles per liter, multiply by 88.4; ALT to microkatals per liter, multiply by 0.0167; D-dimer to nanomoles per liter, multiply by 5.476; creatine kinase to microkatals per liter, multiply by 0.0167; and LDH to microkatals per liter, multiply by 0.0167.

^a^ Linear model was used for comparison of mean values, log-linear model for comparison of median values, and Pearson χ^2^ for categorical variables.

^b^ Short cycle spans 1 to 2 years, medium cycle spans 3.5 to 4 years, and long cycle comprises 5 years, divisible into a 3-year bachelor’s program and a 2-year master’s program.

^c^ Range, 0 to 5 (0, no symptoms at all; 1, no significant disability despite symptoms [able to carry out all usual duties and activities]; 2, slight disability [unable to carry out all previous activities but able to look after own affairs without assistance]; 3, moderate disability [requiring some help but able to walk without assistance]; 4, moderately severe disability [unable to walk without assistance and unable to attend to own bodily needs without assistance]; 5, severe disability [bedridden, incontinent, and requiring constant nursing care and attention]).

^d^ Missing data from 18 hospitalized individuals.

^e^ Missing data from 22 hospitalized individuals.

^f^ Missing data from 1 hospitalized individual.

^g^ Missing data from 55 hospitalized individuals and 1 patient with COVID-19.

^h^ Missing data from 63 hospitalized individuals and 25 patients with COVID-19.

^i^ Missing data from 63 hospitalized individuals and 79 patients with COVID-19.

^j^ Missing values from 43 hospitalized individuals and patients with 5 COVID-19.

### Baseline Characteristics

Patients with COVID-19 had a higher mean (SD) BMI than the control groups (patients with COVID-19, 29.6 [6.0]; hospitalized controls, 26.2 [4.3]; healthy controls, 25.6 [5.2]; *P* < .001) (Table 1). Healthy controls had a higher mean (SD) educational level than patients with COVID-19 (mean [SD], 14.8 [2.7] vs 13.1 [3.1] years) as well as fewer comorbidities (51 [51.0%] vs 92 [76.7%]). There was no significant difference in educational level (mean [SD], 13.1 [3.1] vs 13.4 [2.4] years) and number of comorbidities (92 [76.7%] vs 104 [83.2%]) between patients with COVID-19 and hospitalized controls. The total median number of days of admission was 7.0 (IQR, 4.0-18.0 days) for patients with COVID-19 and 5.0 (IQR, 3.0-14.0 days) for hospitalized controls (*P* = .12). Some controls (43.2% of hospitalized controls [54 of 125] and 54.0% of healthy controls [54 of 100]) had been previously infected with SARS-CoV-2. Peak levels of leukocytes and creatine kinase were lower, and lactate dehydrogenase levels were higher among patients with COVID-19 than hospitalized controls. Mean (SD) time from hospital discharge to follow-up was 542.1 (167.6) days in the COVID-19 group and 640.6 (161.9) days in the control group (Table 1; eTable 2 in Supplement 1). Subgroups are described in eTables 3 and 4 in Supplement 1.

### Primary Outcomes

#### Cognition

**Figure 2.**
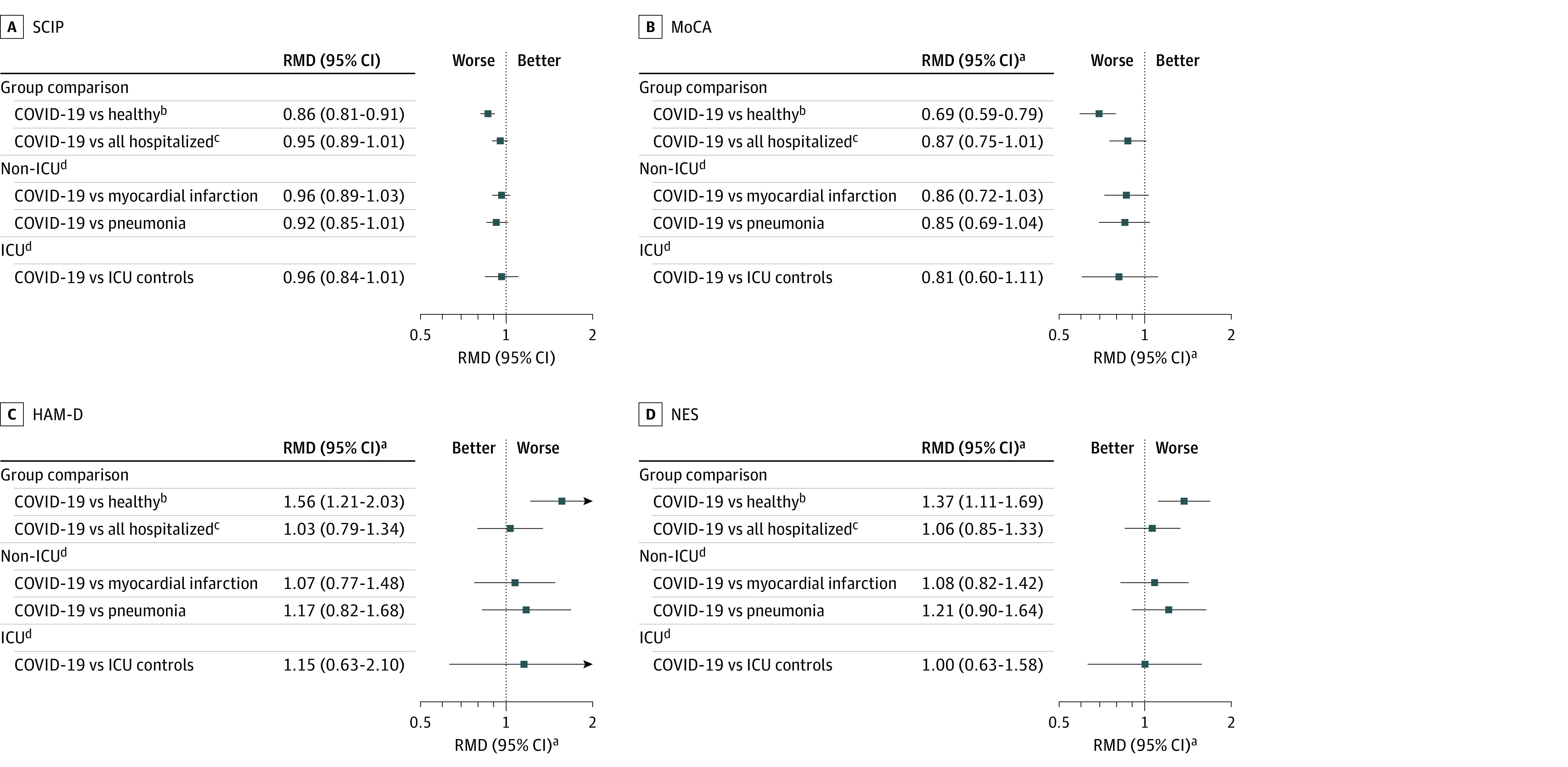
Primary Outcomes and 2 Secondary Outcomes Compared Between Patients With COVID-19 and Control Groups Relative mean difference (RMD) of primary cognitive outcomes and secondary psychiatric and neurological outcomes. See eFigure 2 in Supplement 1 for other secondary outcomes. HAM-D indicates Hamilton Depression Rating Scale; MoCA, the Montreal Cognitive Assessment; NES, Neurological Evaluation Scale; and SCIP, Screen for Cognitive Impairment in Psychiatry. ^a^Relative mean difference for MoCA scores refers to mean difference of 32 – MoCA score and for HAM-D and the NES to total score +1. ^b^Models are adjusted for sex, age, body mass index, educational level, alcohol abuse, smoking, and intensive care unit (ICU) admission. ^c^Models are adjusted for sex, age, admission length, time from hospitalization to follow-up, ICU admission, and disease severity. ^d^Models are adjusted for sex, age, admission length, and time from hospitalization to follow-up.

The estimated mean SCIP score at 18-month follow-up was 59.0 (95% CI, 56.9-61.2) among patients with COVID-19, which was decreased compared with healthy controls (estimated mean SCIP score, 68.8 [95% CI, 66.2-71.5]; *P* < .001) but not significantly decreased compared with hospitalized controls (estimated mean SCIP score, 61.6 [95% CI, 59.1-64.1]; *P* = .12) (Figure 2, Table 2, and Table 3; eTable 5 in Supplement 1). Similarly, the estimated mean MoCA score was 26.5 (95% CI, 26.0-27.0) among the COVID-19 group, which was decreased compared with healthy controls (estimated mean MoCA score, 28.2 [95% CI, 27.8-28.6]; *P* < .001) but not significantly decreased compared with hospitalized controls (estimated mean MoCA score, 27.2 [95% CI, 26.8-27.7; *P* = .07). We also found that 46 of 120 patients with COVID-19 (38.3%) had MoCA scores below 26 at 18-month follow-up.

**Table 2.** Cognitive, Neurological, and Psychiatric Outcomes Compared Between Patients With COVID-19 and All Hospitalized Individuals

Outcome	Estimated mean (95% CI)		Relative mean difference (95% CI)^a^	*P* value^b^
	Patients with COVID-19 (n = 120)	All hospitalized individuals (n = 125)		
Primary outcomes^c^				
SCIP score^d^	58.5 (56.1-61.1)	61.6 (59.1-64.1)	0.95 (0.89-1.01)	.12
MoCA score	26.5 (26.0-27.0)	27.2 (26.8-27.7)	0.87 (0.75-1.01)	.07
Secondary outcomes				
HAM-A	3.7 (3.1-4.4)	2. 8 (2.4-3.4)	1.30 (0.99-1.71)	.06
HAM-D	3.6 (3.1-4.4)	3.5 (3.0-4.2)	1.03 (0.79-1.34)	.83
Neurological Evaluation Scale^e^	8.2 (7.0-9.5)	7.7 (6.7-8.9)	1.06 (0.85-1.33)	.59
Trail Making Test A^f^	36.2 (33.6-39.0)	32.4 (30.1-34.9)	1.12 (1.00-1.23)	.05
Trail Making Test B^g^	92.5 (84.7-101.0)	80.6 (74.1-87.7)	1.15 (1.01-1.31)	.04

Abbreviations: HAM-A, Hamilton Anxiety Rating Scale; HAM-D, Hamilton Depression Rating Scale; MoCA, Montreal Cognitive Assessment; SCIP, Screen for Cognitive Impairment in Psychiatry.

^a^ Relative mean difference for MoCA score refers to mean difference of “32 – MoCA score” and for HAM-A, HAM-D, and the Neurological Evaluation Scale to total score +1.

^b^ Model adjusted for sex, age, admission length, time from hospitalization to follow-up, intensive care unit admission, and severity.

^c^ For description of scores, see eMethods 4 in Supplement 1.

^d^ Missing data on 7 patients with COVID-19 and 1 control patient.

^e^ Missing data on 1 patient with COVID-19.

^f^ Missing data on 1 patient with COVID-19.

^g^ Missing data on 2 patients with COVID-19.

**Table 3.** Cognitive, Neurological, and Psychiatric Outcomes Compared Between Patients With COVID-19 and Healthy Controls

Outcome	Estimated mean (95% CI)		Relative mean difference (95% CI)^a^	*P* value^b^
	COVID-19 patients (n = 120)	Healthy controls (n = 100)		
Primary outcomes^c^				
SCIP score^d^	59.0 (56.9-61.2)	68.8 (66.2-71.5)	0.86 (0.81-0.91)	<.001
MoCA score	26.5 (26.0-27.0)	28.2 (27.8-28.6)	0.69 (0.59-0.79)	<.001
Secondary outcomes				
HAM-A	3.7 (3.1-4.4)	2.5 (2.0-3.0)	1.49 (1.14-1.94)	.003
HAM-D	3.8 (3.2-4.5)	2.5 (2.0-3.0)	1.56 (1.21-2.03)	.001
Neurological Evaluation Scale^e^	7.8 (6.8-8.9)	5.7 (4.9-6.6)	1.37 (1.11-1.69)	.004
Trail Making Test A^f^	36.1 (33.7-38.7)	30.3 (28.1-32.7)	1.19 (1.07-1.33)	.002
Trail Making Test B^g^	90.7 (83.3-98.9)	68.7 (62.5-75.5)	1.32 (1.16-1.51)	<.001

Abbreviations: HAM-A, Hamilton Anxiety Rating Scale; HAM-D, Hamilton Depression Rating Scale; MoCA, Montreal Cognitive Assessment; SCIP, Screening for Cognitive Impairment in Psychiatry.

^a^ Relative mean difference for MoCA scores refers to mean difference of “32 – MoCA score” and for HAM-A, HAM-D, and the Neurological Evaluation Scale to total score +1.

^b^ Models are adjusted for sex, age, body mass index, educational level, alcohol abuse, smoking, and intensive care unit admission and include hospitalized individuals. Full results are presented in eTable 5 in Supplement 1.

^c^ For description of scores, see eMethods 4 in Supplement 1.

^d^ Missing data on 7 patients with COVID-19.

^e^ Missing data on 1 patient with COVID-19.

^f^ Missing data on 1 patient with COVID-19.

^g^ Missing data on 2 patients with COVID-19.

Comparisons between patients with COVID-19 and healthy controls were adjusted for sex, age, BMI, educational level, alcohol abuse, and smoking. Comparisons with hospitalized controls were adjusted for age, sex, ICU admission, total number of admission days, number of days to follow-up, and disease severity. We found similar results with no significant difference in SCIP and MoCA scores when stratifying for ICU admission in models adjusted for age, sex, total number of admission days, and number of days to follow-up (Figure 2; eTables 6 and 7 in Supplement 1).

### Secondary Outcomes

#### Psychiatric Symptom Scales

Among patients with COVID-19, the estimated mean HAM-A score was elevated by a relative mean difference (RMD) of 1.49 (95% CI, 1.14-1.94) compared with healthy controls (*P* = .003) (Figure 2; Table 2 and Table 3; eFigure 2 in Supplement 1). However, there was no significant difference between patients with COVID-19 and hospitalized patietns (RMD, 1.30 [95% CI, 0.99-1.71]; *P* = .06). HAM-D scores were likewise higher in the group with COVID-19 compared with healthy controls (RMD, 1.56 [95% CI, 1.21-2.03]; *P* = .001) but were not significantly higher compared with hospitalized controls (RMD, 1.03 [95% CI, 0.79-1.34]; *P* = .83).

#### Neurological Symptom Scales

The estimated mean score for the Neurological Evaluation Scale was higher (ie, worse performance) among patients with COVID-19 than among healthy controls (RMD, 1.37 [95% CI, 1.11-1.69]; *P* = .004) but was not higher than among hospitalized individuals (RMD, 1.06 [95% CI, 0.85-1.33; *P* = .59) (Figure 2; Table 2 and Table 3).

#### Secondary Cognition Outcomes

Patients with COVID-19 were slower (had higher scores) in completing both Trail Making Tests A and B compared with healthy controls. Trail Making Test A scores were 19% higher (RMD, 1.19 [95% CI, 1.07-1.33]; *P* = .002) and Trail Making Test B scores were 32% higher (RMD, 1.32 [95% CI, 1.16-1.51]; *P* < .001) among patients with COVID-19 (eFigure 2 in Supplement 1; Table 2 and Table 3). However, when comparing patients with COVID-19 and hospitalized controls, only the Trail Making Test B scores were significantly different; patients with COVID-19 had a 15% higher estimated mean score (RMD, 1.15 [95% CI, 1.01-1.31]; *P* = .04).

Results were similar between patients in the ICU and those not in the ICU, with no significant difference between the groups in any of the secondary outcomes (Figure 2; eTables 6 and 7 in Supplement 1). Models followed the same adjustments as for primary outcomes.

### Exploratory Outcomes

Patients with COVID-19 reported a higher frequency of psychiatric and neurological symptoms compared with hospitalized controls (96.7% [116 of 120] vs 83.2% [104 of 125]). However, only anosmia was significantly more frequent at 18-month follow-up after adjusting for multiple testing (eTable 8 in Supplement 1). Similarly, among the neurological signs, after adjusting for multiple testing, only olfactory impairment remained less frequent among healthy controls compared with patients with COVID-19 (16 of 100 [16.0%] vs 46 of 119 [38.7%]; OR for nerve impairment variable, 0.20 [95% CI, 0.10-0.40]; reciprocal OR, 5.0 [95% CI, 2.5-10.0]) (eTable 9 in Supplement 1).

Patients with COVID-19 had a higher incidence of new psychiatric diagnoses compared with healthy controls (OR, 11.24 [95% CI, 4.17-30.31]; *P* < .001) but not compared with control patients after multiplicity adjustment (OR, 2.05 [95% CI, 1.12-3.75]; *P* = .02) (eTable 10 in Supplement 1). In addition, patients with COVID-19 scored higher on the Fatigue Assessment Scale than healthy controls (RMD, 1.48 [95% CI, 1.30-1.68]; *P* < .001) (eTable 11 in Supplement 1).

### Longitudinal Data

Fifty-six patients with COVID-19 were examined 6 months after hospitalization (June 2020 to July 2021) with the MoCA, MINI, objective neurological examination, and symptoms interview. Furthermore, 16 patients with COVID-19 were examined with the MoCA at hospital discharge.^25^ The mean MoCA score among patients with COVID-19 increased between hospital discharge and 18 months of follow-up (21.7 [95% CI, 18.7-23.9] vs 26.5 [95% CI, 25.9-27.0; *P* < .001) but decreased slightly between 6 and 18 months (from 27.4 [95% CI, 26.7-28.0] to 26.5 [95% CI, 25.9-27.0]; *P* = .01) (eFigure 3 and eTable 12 in Supplement 1).

There was an increased frequency from 6 months to 18 months of psychiatric diagnoses assessed using the MINI (from 17.9% [10 of 56] to 32.1% [18 of 56]; OR, 3.00 [95% CI, 0.97-9.28]; *P* = .04) and positive findings during the neurological examination (from 25.0% [14 of 56] to 51.8% [29 of 56]; OR, 4.75 [95% CI, 1.62-13.96]; *P* = .001). See eTable 13 and eFigure 4 in Supplement 1 for subjective symptoms.

### Sensitivity Analyses

We conducted sensitivity analyses on primary outcomes between subgroups of patients in the ICU and not in the ICU. The overall difference between cases and controls remained nonsignificant when adjusted for several additional factors, including sex, age, admission length, time from hospitalization to follow-up, disease severity, educational level, grade, depression, alcohol abuse, smoking, malignant neoplasms, previous medical and psychiatric history, and delirium (eTables 14-17 in Supplement 1).

## Discussion

In this prospective cohort study, patients with COVID-19 performed worse than healthy controls in all cognitive, psychiatric, and neurological tests 18 months after hospitalization. Patients with COVID-19 more often had new psychiatric diagnoses, fatigue, and impaired olfaction than healthy controls. However, patients with COVID-19 had similar outcomes as hospitalized controls, except for executive function and impaired olfaction. In the group with COVID-19, we observed a substantial improvement in cognitive scores between discharge and 18-month follow-up, but not between 6-month and 18-month follow-up. Patients with COVID-19 also experienced an increase in psychiatric comorbidities, neurological findings, and subjective symptoms involving memory and sleep between 6-month and 18-month follow-up.

Previous studies of cognitive function among patients with COVID-19 showed persistent cognitive impairment among 12% to 50% of individuals 1 year after infection.^26,27,28^ We found that 38% of patients with COVID-19 had MoCA scores below 26 at 18-month follow-up and performed worse in all cognitive tests compared with the healthy population, consistent with previous research.^8^ A large study also identified a higher risk of mild cognitive decline among older patients hospitalized with COVID-19 compared with healthy controls.^27^ Our findings showed overall similar cognitive performances between patients with COVID-19 cases and matched controls hospitalized for non–COVID-19 causes. This finding corroborates a 4-month follow-up study comparing a cohort of patients with COVID-19 with a cohort with sepsis, which found no disparity between the cohorts in MoCA scores.^14^ These findings underscore the fact that all these medical conditions are associated with cognitive impairment.^9,11,29^

Corroborating previous studies, we found that anxiety and depression were more frequent among patients with COVID-19 compared with healthy controls but no more frequent than among other hospitalized patients.^7^ In line with 2 retrospective studies,^12,30^ psychiatric diagnoses assessed by the MINI were also comparably prevalent among patients with COVID-19 and hospitalized controls.

As for neurological outcomes, patients with COVID-19 more often had neurological soft signs^31,32^ compared with healthy controls but not compared with hospitalized controls. Underlying factors such as cardiovascular disease, hypertriglyceridemia, and hypertension can cause subtle neurological abnormalities^33^ and may explain the similarity between patients with COVID-19 and controls. During the neurological examination, only impaired olfaction was more common among patients with COVID-19 (38.7%) than healthy controls (16.0%). Neurological abnormalities, including olfactory dysfunction, have been previously reported among patients with COVID-19.^34^ Subjective anosmia was also more frequent among patients with COVID-19, which might be explained by invasion of SARS-CoV-2 in olfactory pathways.^35^

As to longitudinal results, the patients with COVID-19 showed decreasing MoCA scores and increasing subjective memory deficits between follow-up visits. The previous literature is heterogenous, reporting improvement,^7^ unchanged symptoms,^34^ or declines.^27,36^ In particular, cognitive decline was observed between 2 and 12 months after hospitalization in one study,^36^ while another study found an association between long-term cognitive deterioration and COVID-19 among older individuals.^27^ Furthermore, cognitive scores might decrease over time, especially among older populations,^37^ and we did notice increased psychiatric diagnoses (from 17.9% to 32.1%) and neurological abnormalities (from 25.0% to 51.8%) among patients with COVID-19 over time. One explanation could be selection bias, where individuals who chose to participate in both follow-up visits might have had ongoing concerns and desired further evaluation. However, previous studies have reported similar rates of psychiatric morbidity (45%)^26^ and neurological abnormalities (64%)^34^ 1 year after infection. Altogether, while it is essential to interpret these findings with caution due to the limited longitudinal data available, the possibility of worsening brain health over time cannot be ruled out.

### Limitations

This study has some limitations. First, the follow-up period was relatively long, which could have affected the observed frequency of some outcome measures. Second, the groups were slightly different at baseline, with the hospitalized group being older and healthy controls having relatively fewer comorbidities and overall higher educational levels than patients with COVID-19. Nonetheless, our results remained unchanged when adjusted for age, comorbidities, and educational level. Third, the inclusion period covered different strains of SARS-CoV-2 with varying virulence and potential for long-term effects. Fourth, some controls (43.2% of hospitalized controls and 54.0% of healthy controls) had been previously infected with SARS-CoV-2. However, these individuals had not been hospitalized for COVID-19, and their infections had been mild or asymptomatic and occurred at least 3 months prior to study enrollment. Fifth, we included the SCIP test, which has no ceiling effect and covers all the most relevant cognitive domains, but the cognitive test battery was comparably small. Sixth, for obvious reasons, cognitive scores from before the pandemic were unavailable, which precludes precise quantification of brain health deficits compared with premorbid conditions, even though we excluded people with known preexisting cognitive impairment.

## Conclusions

This cohort study suggests that patients hospitalized with COVID-19 had worse cognitive, neurological, and psychiatric outcomes at 18-month follow-up than healthy controls. However, compared with carefully matched patients requiring hospitalization for pneumonia, myocardial infarction, or non–COVID-19, ICU-requiring illness, there were no statistically significant differences. Because healthy controls had fewer comorbidities than hospitalized individuals, we conclude that multimorbidity plays a role in both hospitalization and lasting associations with brain health. Although studies with broader cognitive test batteries are needed to confirm these findings, brain health after COVID-19 seems overall comparable to that after other diseases of similar severity.

## Notes

Supplement 1. **eMethods 1.** Inclusion and Exclusion Criteria (Methods) **eMethods 2.** Characteristics of Control Groups **eMethods 3.** Questionnaire on Subjective Symptoms (Methods) **eMethods 4.** Outcome Measures and Methods of Assessment (Methods) **eTable 1.** ICU Controls Admission Causes **eTable 2.** Baseline Characteristics of COVID-19, All Hospitalized Individuals, and Healthy Controls **eTable 3.** Baseline Characteristics of COVID-19 Non-ICU, Pneumonia, Myocardial Infarction and Healthy Controls **eTable 4.** Baseline Characteristics of COVID-19 ICU and ICU Controls **eTable 5.** Cognitive, Neurological, and Psychiatric Outcomes Compared Between COVID-19 Patients, All Hospitalized Individuals and Healthy Controls **eTable 6.** Cognitive, Neurological, and Psychiatric Outcomes Compared Between Non-ICU COVID-19 Patients and the Other Non-ICU Groups in Models Adjusted for Age and Sex (A) and Fully Adjusted (B) **eTable 7.** Cognitive, Neurological, and Psychiatric Outcomes Compared Between ICU COVID-19 and ICU Controls in Models Adjusted for Age and Sex (A) and Fully Adjusted (B) **eTable 8.** Self-Reported Neuropsychiatric Symptoms in COVID-19 Patients and Hospitalized Controls at the 18 Months Investigation **eTable 9.** Neurological Examination Findings in COVID-19 Patients, Hospitalized Controls and Healthy Controls at the 18 Months Investigation **eTable 10.** New Onset Psychiatric Disorders Assessed With the MINI Interview in COVID-19 Patients, Hospitalized Controls and Healthy Controls at the 18 Months Investigation **eTable 11.** Fatigue Assessment Scale Scores Compared Between COVID-19 Patients, Hospitalized Controls and Healthy Controls **eTable 12.** Changes in Mean MoCA Score Over Time in COVID-19 Individuals **eTable 13.** Changes in Frequency of Neurological Findings, Psychiatric Diagnoses and Subjective Symptoms in COVID-19 Individuals Between 6- and 18-Months Follow-Up Visits **eTable 14.** Sensitivity Analyses for SCIP Scores in Non-ICU COVID-19 Patients and Non-ICU Hospitalized Controls **eTable 15.** Sensitivity Analyses for SCIP Scores in ICU COVID-19 and ICU Controls **eTable 16.** Sensitivity Analyses for MoCA Scores in Non-ICU COVID-19 Patients and Non-ICU Hospitalized Controls **eTable 17.** Sensitivity Analyses for MoCA Scores in ICU COVID-19 and ICU Controls **eFigure 1.** Detailed Flowchart of Inclusion Process **eFigure 2.** Secondary Outcomes Compared Between COVID-19 and Control Groups **eFigure 3.** MoCA Scores in COVID-19 Cases and Controls at 18-Month Follow-Up and Changes in Mean MoCA Scores Over Time in COVID-19 Cases **eFigure 4.** Radar Chart Showing Trajectories of Psychiatric Symptoms and Diagnoses (A) and Neurological Symptoms and Signs (B) From 6 to 18 Months After COVID-19 Hospitalization
eReferences.

Supplement 2.
Data Sharing Statement
