# Supplementary material for: Brain Health After COVID-19, Pneumonia, Myocardial Infarction, or Critical Illness
Source: JAMA Netw Open. 2023 Dec 28;6(12):e2349659. doi: 10.1001/jamanetworkopen.2023.49659 (PMC10755623; doi:10.1001/jamanetworkopen.2023.49659)
Supplement: Supplement 1. — eMethods 1. Inclusion and Exclusion Criteria (Methods) eMethods 2. Characteristics of Control Groups eMethods 3. Questionnaire on Subjective Symptoms (Methods) eMethods 4. Outcome Measures and Methods of Assessment (Methods) eTable 1. ICU Controls Admission Causes eTable 2. Baseline Characteristics of COVID-19, All Hospitalized Individuals, and Healthy Controls eTable 3. Baseline Characteristics of COVID-19 Non-ICU, Pneumonia, Myocardial Infarction and Healthy Controls eTable 4. Baseline Characteristics of COVID-19 ICU and ICU Controls eTable 5. Cognitive, Neurological, and Psychiatric Outcomes Compared Between COVID-19 Patients, All Hospitalized Individuals and Healthy Controls eTable 6. Cognitive, Neurological, and Psychiatric Outcomes Compared Between Non-ICU COVID-19 Patients and the Other Non-ICU Groups in Models Adjusted for Age and Sex (A) and Fully Adjusted (B) eTable 7. Cognitive, Neurological, and Psychiatric Outcomes Compared Between ICU COVID-19 and ICU Controls in Models Adjusted for Age and Sex (A) and Fully Adjusted (B) eTable 8. Self-Reported Neuropsychiatric Symptoms in COVID-19 Patients and Hospitalized Controls at the 18 Months Investigation eTable 9. Neurological Examination Findings in COVID-19 Patients, Hospitalized Controls and Healthy Controls at the 18 Months Investigation eTable 10. New Onset Psychiatric Disorders Assessed With the MINI Interview in COVID-19 Patients, Hospitalized Controls and Healthy Controls at the 18 Months Investigation eTable 11. Fatigue Assessment Scale Scores Compared Between COVID-19 Patients, Hospitalized Controls and Healthy Controls eTable 12. Changes in Mean MoCA Score Over Time in COVID-19 Individuals eTable 13. Changes in Frequency of Neurological Findings, Psychiatric Diagnoses and Subjective Symptoms in COVID-19 Individuals Between 6- and 18-Months Follow-Up Visits eTable 14. Sensitivity Analyses for SCIP Scores in Non-ICU COVID-19 Patients and Non-ICU Hospitalized Controls eTable 15. Sensitivity Analyses fo [file jamanetwopen-e2349659-s001.pdf]

Supplementary Online Content

Peinkhofer C, Zarifkar P, Christensen RHB, et al. Brain health after COVID-19, pneumonia, myocardial infarction, or critical illness. *JAMA Netw Open.* 2023;6(12):e2349659. doi:10.1001/jamanetworkopen.2023.49659

eMethods 1. Inclusion and Exclusion Criteria (Methods)
eMethods 2. Characteristic of Control Groups
eMethods 3. Questionnaire on Subjective Symptoms (Methods)
eMethods 4. Outcome Measures and Methods of Assessment (Methods)
eTable 1. ICU Controls Admission Causes
eTable 2. Baseline Characteristics of COVID-19, All Hospitalized Individuals, and Healthy Controls
eTable 3. Baseline Characteristics of COVID-19 Non-ICU, Pneumonia, Myocardial Infarction and Healthy Controls
eTable 4. Baseline Characteristics of COVID-19 ICU and ICU Controls
eTable 5. Cognitive, Neurological, and Psychiatric Outcomes Compared Between COVID-19 Patients, All Hospitalized Individuals and Healthy Controls
eTable 6. Cognitive, Neurological, and Psychiatric Outcomes Compared Between Non-ICU COVID-19 Patients and the Other Non-ICU Groups in Models Adjusted for Age and Sex (A) and Fully Adjusted (B)
eTable 7. Cognitive, Neurological, and Psychiatric Outcomes Compared Between ICU COVID-19 and ICU Controls in Models Adjusted for Age and Sex (A) and Fully Adjusted (B)
eTable 8. Self-Reported Neuropsychiatric Symptoms in COVID-19 Patients and Hospitalized Controls at the 18 Months Investigation
eTable 9. Neurological Examination Findings in COVID-19 Patients, Hospitalized Controls and Healthy Controls at the 18 Months Investigation
eTable 10. New Onset Psychiatric Disorders Assessed With the MINI Interview in COVID-19 Patients, Hospitalized Controls and Healthy Controls at the 18 Months Investigation
eTable 11. Fatigue Assessment Scale Scores Compared Between COVID-19 Patients, Hospitalized Controls and Healthy Controls
eTable 12. Changes in Mean MoCA Score Over Time in COVID-19 Individuals
eTable 13. Changes in Frequency of Neurological Findings, Psychiatric Diagnoses and Subjective Symptoms in COVID-19 Individuals Between 6- and 18-Months Follow-up Visits
eTable 14. Sensitivity Analyses for SCIP Scores in Non-ICU COVID-19 Patients and Non-ICU Hospitalized Controls
eTable 15. Sensitivity Analyses for SCIP Scores in ICU COVID-19 and ICU Controls
eTable 16. Sensitivity Analyses for MoCA Scores in Non-ICU COVID-19 Patients and Non-ICU Hospitalized Controls
eTable 17. Sensitivity Analyses for MoCA Scores in ICU COVID-19 and ICU Controls
eFigure 1. Detailed Flowchart of Inclusion Process
eFigure 2. Secondary Outcomes Compared Between COVID-19 and Control Groups
eFigure 3. MoCA Scores in COVID-19 Cases and Controls at 18-Month Follow-up and Changes in Mean MoCA Scores Over Time in COVID-19 Cases
eFigure 4. Radar Chart Showing Trajectories of Psychiatric Symptoms and Diagnoses (A) and Neurological Symptoms and Signs (B) From 6 to 18 Months After COVID-19 Hospitalization
eReferences.

This supplementary material has been provided by the authors to give readers additional information about their work.

© 2023 Peinkhofer C et al. *JAMA Network Open.*

eMethods 1. Inclusion and Exclusion Criteria (Methods)

All participants were ≥ 18 years old and had signed informed written consent.

Hospitalized COVID-19 participants (group 1):
Inclusion criteria:

- Manifest COVID-19 infection requiring hospitalization ≥ 24h (ICU and non-ICU units)
- Verified SARS-CoV-2 PCR by nasopharyngeal/tracheal testing during hospitalization

Hospitalized control participants (groups 2,3,4)
- Pneumonia or myocardial infarction requiring hospitalization ≥ 24h or
- Admission to the ICU ≥ 4 days and with a total admission length ≥ 10 days for any reason besides COVID-19 infection or myocardial infarction

Healthy control participants (5)
Inclusion criteria:
- No hospitalization in the last year and no recent (<3 months) COVID-19 infection.

All groups exclusion criteria
Exclusion criteria:
- Residency outside of Denmark
- Lack of Danish or English language proficiency
- ICU admission during the follow-up period
- Any pre-existing neurologic or psychiatric disorders that would confound follow-up for new-onset cognitive and mental health complications, including congenital intellectual disability, a history of dementia, severe CNS injury requiring neurorehabilitation and recent (<1 year) debut of psychosis

Additional exclusion criteria for individual groups:
Group 2
- Hospitalization for pneumonia or COVID-19
- Multiple STEMI or CABG

Group 3
- Hospitalization for STEMI or COVID-19
- Multiple admissions for pneumonia

© 2023 Peinkhofer C et al. *JAMA Network Open.*

eMethods 2. Characteristic of Control Groups

Control groups consisted of:

(a) *healthy volunteers*: ≥ 18 years old and without hospitalization in the preceding 2 years, recruited via local newspapers and the internet;

(b) *patients hospitalized for three non-COVID causes* (i.e., pneumonia, acute myocardial infarction, and non-COVID ICU-requiring illness). The control patient cohorts included:

(i) patients admitted to Gentofte Hospital between March 2020 and June 2021 with pneumonia but no history of myocardial infarction;

(ii) patients admitted to Rigshospitalet between September 2020 and March 2021 with acute myocardial infarction who had percutaneous coronary intervention performed and no history of pneumonia and no ICU admission;

(iii) patients admitted to Rigshospitalet's medical and surgical ICUs between March 2020 and January 2021 for reasons other than COVID-19 (eTable1)

© 2023 Peinkhofer C et al. *JAMA Network Open.*

eMethods 3. Questionnaire on Subjective Symptoms (Methods)

1.Have you experienced changes in you smell sense?<br>If yes, is your smell sense still altered now?	Yes/no<br>No/partially/completely
2.Have you experienced any olfactory hallucinations?	Yes/no
3.Have you experienced changes in your taste function?<br>If yes, are you currently experiences any problems?	Yes/no<br>No/partially/completely
4.Have you experienced any taste hallucinations?	Yes/no
5.Have you noticed changed in your vision?	Yes/no
6.Are you experiencing paresthesia?	No/mild/moderate/ severe
7.Are you having difficulties with your short-term memory?	No/mild/moderate/ severe
8.Are you having difficulties with your long-term memory?	No/mild/moderate/ severe
9.Are you experiencing concertation problems?	No/mild/moderate/ severe
10.Are you experiencing any speech difficulties (finding the right words or making more mistakes while talking)?	No/mild (sometimes) /moderate (often) / severe (daily)
11.Do you have any new-onset pain?	No/mild/moderate/severe
12.Are you experiencing a new form for headache (after hospitalization) or worsening of a previous headache?	Yes, worsening/yes, new onset/no
13.Are you experiencing any dizziness? (not positional)	No/mild/moderate/ severe
14.Do you have tinnitus?	No/mild/moderate/ severe
15.Have you experienced disturbing flashbacks or nightmares after hospitalization?	No/mild (sometimes) /moderate (often)/ severe (daily)

© 2023 Peinkhofer C et al. *JAMA Network Open.*

16.Have you needed psychological help after hospitalization?	No/ short (<1 year)/long (> 1 year)
17.Have you felt isolated or lonely during the pandemic period?	No/mild/moderate/severe

© 2023 Peinkhofer C et al. *JAMA Network Open.*

eMethods 4. Outcome Measures and Methods of Assessment (Methods)

**Outcome measures**	**Assessment method**
**Primary outcomes**	
Cognition	Montreal Cognitive Assessment (MoCA), Danish version. MoCA tests memory, executive functioning, attention, language, visuospatial, orientation. Maximum score is 30 and cognitive impairment is indicated by a score ≤ 26.<br><br>Screening for Cognitive Impairment in Psychiatry (SCIP) in Danish. SCIP tests verbal learning, working memory, verbal fluency, delayed recall, and psychomotor speed impairments. There is no maximum score and < 75 indicates mild impairment mild cognitive impairment.
**Secondary outcomes**	
Depression and anxiety symptoms	Hamilton Anxiety Scale measures anxiety symptoms and is based 14 items. Each item is scored on a scale from 0 (not present) to 4 (severe). Total score rages from 0-56 and a score <17 indicates mild anxiety, 18–24 mild to moderate and 25–30 moderate to severe.<br><br>Hamilton Depression scale measures depression symptoms and is based on 17 items. Each item is scored on a scale from 0 (not present) to 4 (severe). Total score ranges from 0-52 and a score of 10 – 13 indicates mild depression, 14-17 mild to moderate, >17 moderate to severe.
Neurological function	Neurological Evaluation Scale (NES) is used to assess neurological soft signs. It is composed of 26 items testing sensory integration, motor coordination, complex motor acts and other functions such as primitive reflexes. Higher scores indicate worse performance. Items are rated on a 3-point scale: 0 = no abnormality; 1 = mild, but definite impairment; and 2 = marked impairment, except for the snout and suck reflexes which are scored either as a 0 or 2.
Cognition	Trail Making Test A(numeric) and B (alphanumeric) evaluate executive function. The longer it takes to complete the test, the lower the score. An average score for TMT-A is 29 seconds and a deficient score is greater than 78 seconds. For TMT-B an average score is 75 seconds, and a deficient score is greater than 273 seconds.
Exploratory outcomes	
Neurological function	Neurologic examination´s findings including cranial nerves, sensorimotor and cerebellar functions, including reflexes, muscle strength, coordination, stance, gait, and primitive reflexes.<br><br>4-item pocket smell test, “scratch and sniff” assessed olfactory nerve function. A score ≤ 3 indicates possible dysfunction and the necessity of further investigation.
New onset psychiatric diagnoses	Mini International Neuropsychiatric Interview (MINI), version 5.0 in Danish. The MINI is a brief structured diagnostic interview for assessing 17 of the most common psychiatric disorders and is based on yes/no questions. We selected the following disorders: depression, suicide, hypomania and mania, panic anxiety with and without agoraphobia, agoraphobia without panic anxiety, social phobia, OCD, PTSD and psychosis.
Persistent neuropsychiatric symptoms	Semi-structured interview consisting of 17 questions for self-assessment of persisting symptoms. Some symptoms are rated in terms of severity (mild/moderate/severe; daily/often/sometimes/never) and others in terms of presence (yes/no).
Fatigue	The Fatigue Assessment Scale is a 10 items scale to assess the levels of fatigue. Items are rated from 0 to 5 (never, sometimes, regularly, often, always). Total score ranges from 10 to 50. A total FAS score < 22 indicates no fatigue, a score ≥ 22 indicates fatigue.
Change over time	MoCA scores, presence of any neurological abnormality at neurological examination, presence of any psychiatric diagnosis assessed via MINI and frequency of subjective symptoms assessed with a semi-structured interview.

© 2023 Peinkhofer C et al. *JAMA Network Open.*

**eTable 1.** ICU Controls Admission Causes	
**Intensive care unit admission causes**	**N. of patients**
Abdominal vascular complication/trauma	2
Multitrauma	2
Organ-failure/infection of unknown etiology	2
Pharyngeal/laryngeal infection with respiratory complications	3
Post-surgery complications (e.g. sepsis, respiratory or kidney failure etc.)	6
Ruptured abdominal aortic aneurysm 6	5
Sepsis with multiorgan affection	3
Thoracic aneurysm/varicose veins with hemoptysis	2

© 2023 Peinkhofer C et al. *JAMA Network Open.*

eTable 2. Baseline Characteristics of COVID-19, All Hospitalized Individuals, and Healthy Controls

	COVID-19 (n=120)	Hospitalized controls (n=125)	Healthy controls (n=100)	Covid-19 vs hospitalized controls p value[a]	COVID-19 vs healthy controls p value[a]
**Previous medical history**					
COPD	6 (5.0)	9 (7.2)	1 (1.0)	.47	.09
Interstitial lung disease	0 (0.0)	3 (2.4)	0 (0.0)	.09	NA
Liver or pancreas	0 (0.0)	2 (1.6)	0 (0.0)	.16	NA
Gastrointestinal	12 (10.0)	5 (4.0)	2 (2.0)	.07	NA
Gynecology	3 (2.5)	1 (0.8)	0(0.0)	.29	NA
Urology	8 (6.7)	8 (6.4)	2(2.0)	.93	NA
Orthopedic	6 (5.0)	12 (9.6)	0 (0.0)	.17	.02
Nephrology	4 (3.3)	2 (1.6)	0 (0.0)	.38	.07
Major surgery in the last year	3 (2.5)	4 (3.2)	0 (0.0)	.74	.11
Chronic infection	2 (1.7)	3 (2.4)	0 (0.0)	.69	.20
Other cardiac comorbidities	23 (19.2)	35 (28.0)	13 (13.0)	.10	.22
**Previous neurological history**					
Any neurological comorbidity	22 (18.3)	20 (16.0)	8 (8)	.63	.03
Traumatic brain injury	1 (0.8)	0 (0.0)	1 (1.0)	.31	.90
Migraine	4 (3.3)	3 (2.4)	2 (2.0)	.66	.55
Headache other	2 (1.7)	0 (0.0)	0 (0.0)	.15	.20
Epilepsy	1 (0.8)	1 (0.8)	0 (0.0)	.98	.36
Parkinson	1 (0.8)	2 (1.6)	0 (0.0)	.59	.36
Movement disorders	0 (0.0)	0 (0.0)	1 (1.0)	NA	.27
Multiple Sclerosis	0 (0.0)	0 (0.0)	0 (0.0)	NA	NA
Neuromuscular	2 (1.7)	0 (0.0)	0(0.0)	.15	.20
Bone marrow injury	1 (0.8)	0 (0.0)	0 (0.0)	.31	.36
Stroke	4 (3.3)	9 (7.2)	4 (4.0)	.18	.79
Subarachnoid hemorrhage	1 (0.8)	2 (1.6)	0 (0.0)	.59	.36
Benign brain tumor	0 (0.0)	1 (0.8)	0 (0.0)	.33	NA
Neuroinfections	1 (0.8)	2 (1.6)	0 (0.0)	.59	.36
Other neurological comorbidities	12 (10.0)	7 (5.6)	3 (3.0)	.20	.04
**Previous psychiatric history**					
Bipolar	1 (0.8)	0 (0.0)	0 (0.0)	.31	.36
Schizophrenia	0 (0.0)	0 (0.0)	0 (0.0)	NA	NA
OCD	1 (0.8)	1 (0.8)	1 (1.0)	.98	.90
Alcohol abuse	2 (1.7)	5 (4.0)	4 (4.0)	.27	.29
Drug abuse	2 (1.7)	0 (0.0)	1 (1.0)	.15	.67
Eating disorder	1 (0.8)	1 (0.8)	0 (0.0)	.98	.36
Other psychiatric comorbidities	3 (2.5)	6 (4.8)	2 (2.0)	.34	.80
Relatives with psychiatric disorders				.29	.83
Present	31 (25.8)	22 (17.6)	27 (27.0)		
Unknown	4 (3.3)	5 (4.0)	2 (2.0)		
**Before hospitalization**					
Immunosuppression	10 (8.3)	7 (5.6)	0 (0.0)	.40	.003
mRS before hospitalization (SD)	0.2 (0.6)	0.2 (0.5)	NA	.83	NA
Time from COVID-19 symptoms to hospitalization (SD)	8.7 (5.2)	NA	NA	NA	NA
**Admission characteristics**					
Delirium	15 (12.5)	18 (14.4)	NA	.66	NA
Haloperidol	12 (80.0)	12 (66.7)	NA	.39	NA
Olanzapine	2 (13.3)	6 (35.3)	NA	.15	NA

© 2023 Peinkhofer C et al. *JAMA Network Open.*

Other	1 (6.7)	2 (11.8)	NA	.62	NA
Dialysis	8 (27.6)	7 (28.0)	NA	.97	NA
Inotropic agents	19 (65.5)	20 (80.0)	NA	.24	NA
Prone ventilation	23 (19.2)	0 (0.0)	NA	< .001	NA
ARDS	23 (19.2)	0 (0.0)	NA	< .001	NA
**Laboratory findings, median (IQR)**					
Leukocytes (at admission)	6.2 (4.9, 7.8)	10.9 (8.1, 14.1)	NA	< .001	NA
Missing	0	23	NA		NA
Leukocytes (at discharge)	7.0 (5.8, 10.4)	7.9 (6.5, 10.1)	NA	.60	NA
Missing	0	19	NA		NA
CRP (at admission)	72.5 (40.8, 150.2)	49.0 (8.0, 180.0)	NA	.01	NA
Missing	0	28	NA		NA
CRP (at discharge)	14.5 (5.0, 36.2)	25.0 (11.8, 50.0)	NA	< .001	NA
Missing	0	21	NA		NA
Creatinine (at admission)	82.0 (69.0, 99.0)	79.0 (64.0, 93.0)	NA	.01	NA
Missing	0	2	NA		NA
Creatinine (at discharge)	70.0 (59.8, 87.0)	72.0 (60.5, 83.0)	NA	< .001	NA
Missing	0	6	NA		NA
ALAT (at admission)	35.0 (23.5, 59.5)	24.5 (18.0, 43.5)	NA	.06	NA
Missing	1	45	NA		NA
ALAT (at discharge)	48.0 (28.0, 85.0)	44.0 (24.5, 64.0)	NA	.40	NA
Missing	3	69	NA		NA
**Paraclinical characteristics**					
EEG	5 (4.2)	3 (2.4)	NA	.44	NA
Normal	0 (0.0)	1 (0.8)	NA	.33	NA
FIRDA	2 (1.7)	0 (0.0)	NA	.15	NA
Sharp waves	1 (0.8)	0 (0.0)	NA	.31	NA
Encephalopathy	2 (1.7)	2 (1.6)	NA	.97	NA
CT	11 (9.2)	12 (9.6)	NA	.91	NA
Normal	6 (5.0)	7 (5.6)	NA	.84	NA
Old stroke	2 (1.7)	1 (0.8)	NA	.54	NA
Leukoaraiosis	0 (0.0)	2 (1.6)	NA	.16	NA
New hemorrhagic stroke	1 (0.8)	0 (0.0)	NA	.31	NA
SAH	2 (1.7)	0 (0.0)	NA	.15	NA
Other	0 (0.0)	2 (1.6)	NA	.16	NA
MRI	8 (6.7)	3 (2.4)	NA	.11	NA
Normal	3 (2.5)	1 (0.8)	NA	.29	NA
Old stroke	1 (0.8)	1 (0.8)	NA	.98	NA
Leukoaraiosis	1 (0.8)	0 (0.0)	NA	.31	NA
New hemorrhagic stroke	1 (0.8)	0 (0.0)	NA	.31	NA
Hydrocephalus	1 (0.8)	0 (0.0)	NA	.31	NA
SAH	1 (0.8)	0 (0.0)	NA	.31	NA
Other	1 (0.8)	1 (0.8)	NA	.98	NA
Nerve conduction studies	3 (2.5)	1 (0.8)	NA	.29	NA
Normal	2 (1.7)	0 (0.0)	NA	.15	NA
Critical illness neuropathy	0 (0.0)	1 (0.8)	NA	.33	NA
Critical illness myopathy	1 (0.8)	0 (0.0)	NA	.31	NA
Lumbar puncture	6 (5.0)	4 (3.2)	NA	.48	NA
Pleocytosis	1 (0.8)	1 (0.8)	NA	.98	NA

Abbreviations: ALAT = Alanine aminotransferase; BMI = Body mass index; COPD = Chronic obstructive pulmonary disease; CRP = C-reactive protein; EEG = Electroencephalogram; FIRDA = Frontal intermittent rhythmic delta activity; LDH= Lactate dehydrogenase; mRS = modified Rankin scale; OCD = Obsessive compulsive disorder; PTSD = Post-traumatic stress disorder; SAH = Subarachnoid hemorrhage

© 2023 Peinkhofer C et al. *JAMA Network Open.*

Data are presented as n (%), mean (SD), median [IQR]
a Linear model was used for comparison of means; log-linear model for comparison of medians; Pearson´s chi-squared for categorical variables

© 2023 Peinkhofer C et al. *JAMA Network Open*.

eTable 3. Baseline Characteristics of COVID-19 Non-ICU, Pneumonia, Myocardial Infarction and Healthy Controls

	Covid-19 non-ICU (n=91)	Myocardial infarction (n=50)	Pneumonia (n=50)	Healthy (n=100)	p value[a]
**Demographics**					
Age, mean (SD)	60.5 (15.1)	63.0 (11.3)	69.4 (11.6)	62.9 (15.3)	.005
Sex					.04
Female	39 (42.9)	15 (30.0)	25 (50.0)	54 (54.0%)	
Male	52 (57.1)	35 (70.0)	25 (50.0)	46 (46.0%)	
BMI, mean (SD)	29.1 (6.0)	27.4 (4.5)	25.0 (3.7)	25.6 (5.2)	< .001
Smoking					< .001
Current	1 (1.1)	13 (26.0)	2 (4.0)	9 (9.0%)	
Never	46 (50.5)	14(28.0)	21 (42.0)	53 (53.0%)	
Previous	44 (48.4)	23 (46.0)	27 (54.0)	38 (38.0%)	
Education					
Length, mean (SD)	13.2 (3.2)	13.2 (2.4)	13.7 (2.4)	14.8 (2.7)	< .001
Grade					< .001
Primary school	23 (25.3)	7 (14.0)	6 (12.0)	9 (9.0%)	
Vocational training	19 (20.9)	13 (26.0)	12 (24.0)	23 (23.0%)	
Short cycle	11 (12.1)	14 (28.0)	7 (14.0)	4 (4.0%)	
Medium cycle	23 (25.3)	10 (20.0)	18 (36.0)	34 (34.0%)	
Long cycle	15 (16.5)	6 (12.0)	7 (14.0)	30 (30.0%)	
Covid-19 infection	91 (100.0)	30 (60.0)	19 (38.0)	54 (54.0%)	< .001
Covid-19 vaccination	85 (93.4)	49 (98.0)	50 (100.0)	98 (98.0%)	0.12
**Previous medical history**					
Any comorbidity	69 (75.8)	41 (82.0)	43 (86.0)	51 (51.0%)	< .001
Hypertension	31 (34.1)	18 (36.0)	23 (46.0)	28 (28.0%)	.18
Hyperlipidemia	24 (26.4)	28 (56.0)	16 (32.0)	15 (15.0%)	< .001
Diabetes Mellitus type II	16 (17.6)	2 (4.0)	4 (8.0)	5 (5.0%)	.01
Malignancy	12 (13.2)	1 (2.0)	5 (10.0)	9 (9.0%)	.18
Autoimmune disorder	10 (11.0)	7 (14.0)	8 (16.0)	2 (2.0%)	.01
Asthma	16 (17.6)	5 (10.0)	10 (20.0)	3 (3.0%)	.003
Other comorbidities	28 (30.8)	14 (28.0)	21 (42.0)	9 (9.0%)	<.001
COPD	6 (6.6)	0 (0.0)	6 (12.0)	1 (1.0%)	.005
Interstitial lung disease	0 (0.0)	0 (0.0)	3 (6.0)	0 (0.0%)	.002
Liver or pancreas	0 (0.0)	1 (2.0)	0 (0.0)	0 (0.0%)	.18
Gastrointestinal	10 (11.0)	2 (4.0)	2 (4.0)	2 (2.0%)	.05
Gynecology	2 (2.2)	0 (0.0)	0 (0.0)	0 (0.0%)	.22
Urology	7 (7.7)	3 (6.0)	3 (6.0)	2 (2.0%)	.34
Orthopedic	5 (5.5)	2 (4.0)	5 (10.0)	0 (0.0%)	.21
Nephrology	3 (3.3)	1 (2.0)	1 (2.0)	0 (0.0%)	.37
Major surgery in the last year	2 (2.2)	1 (2.0)	1 (2.0)	0 (0.0%)	.54
Chronic infection	2 (2.2)	0 (0.0)	3 (6.0)	0 (0.0%)	.04
Other cardiac comorbidities	19 (20.9)	11 (22.0)	17 (34.0)	13 (13.0%)	.03
**Previous neurological history**					
Any neurological comorbidity	17 (18.7)	5 (10.0)	10 (20.0)	8 (8.0%)	.08
Traumatic brain injury	0 (0.0)	0 (0.0)	0 (0.0)	1 (1.0%)	.59
Migraine	3 (3.3)	1 (2.0)	1 (2.0)	2 (2.0%)	.93
Headache other	2 (2.2)	0 (0.0)	0 (0.0)	0 (0.0%)	.22
Epilepsy	1 (1.1)	0 (0.0)	1 (2.0)	0 (0.0%)	.47
Parkinson	0 (0.0)	0 (0.0)	1 (2.0)	0 (0.0%)	.81

© 2023 Peinkhofer C et al. *JAMA Network Open.*

Movement disorders	0 (0.0)	0 (0.0)	0 (0.0)	1 (1.0%)	.59
Multiple Sclerosis	0 (0.0)	0 (0.0)	0 (0.0)	0 (0.0%)	NA
Neuromuscular	2 (2.2)	0 (0.0)	0 (0.0)	0 (0.0%)	.22
Bone marrow injury	1 (1.1)	0 (0.0)	0 (0.0)	0 (0.0%)	.53
Stroke	1 (1.1)	1 (2.0)	5 (10.0)	4 (4.0%)	.06
Subarachnoid hemorrhage	0 (0.0)	0 (0.0)	1 (2.0)	0 (0.0%)	.18
Benign brain tumor	0 (0.0)	0 (0.0)	1 (2.0)	0 (0.0%)	.18
Neuroinfections	1 (1.1)	1 (2.0)	1 (2.0)	0 (0.0%)	.58
Other neurological comorbidities	9 (9.9)	2 (4.0)	5 (10.0)	3 (3.0%)	.16
**Previous psychiatric history**					
Any psychiatric comorbidity	31 (34.1)	16 (32.0)	13 (26.0)	27 (27.0%)	.65
Depression	17 (18.7)	9 (18.0)	8 (16.0)	13 (13.0%)	.73
Anxiety	8 (8.8)	4 (8.0)	3 (6.0)	11 (11.0%)	.78
PTSD	2 (2.2)	2 (4.0)	1 (2.0)	1 (1.0%)	.68
Stress	5 (5.5)	5 (10.0)	5 (10.0)	4 (4.0%)	.36
Bipolar	1 (1.1)	0 (0.0)	0 (0.0)	0 (0.0%)	.53
Schizophrenia	0(0.0)	0(0.0)	0(0.0)	0(0.0%)	NA
OCD	1 (1.1)	0 (0.0)	1 (2.0)	1 (1.0%)	.81
Alcohol abuse	2 (2.2)	1 (2.0)	1 (2.0)	4 (4.0%)	.83
Drug abuse	1 (1.1)	0 (0.0)	0 (0.0)	1 (1.0%)	.79
Eating disorder	0 (0.0)	1 (2.0)	0 (0.0)	0 (0.0%)	.18
Other psychiatric comorbidities	2 (2.2)	2 (4.0)	3 (6.0)	2 (2.0%)	.53
Relatives with psychiatric disorders					.18
Present	27 (29.7)	12 (24.0)	6 (12.0)	27 (27.0%)	
Unknown	1 (1.1)	3 (6.0)	2 (4.0)	2 (2.0%)	
**Before hospitalization**					
Immunosuppression	8 (8.8)	0 (0.0)	4 (8.0)	0 (0.0%)	.004
mRS before hospitalization (SD)	0.2 (0.6)	0.1 (0.3)	0.3 (0.7)	NA	.07
Time from COVID-19 symptoms to hospitalization (SD)	8.9 (5.5)	NA	NA	NA	
**Admission characteristics**					
Admission days, median (IQR)	5.0 (3.0-8.0)	3.0 (3.0-4.0)	6.0 (4.0-9.0)	NA	.004
Severity scale					< .001
3: Not requiring oxygen	26 (28.6)	46 (92.0)	24 (48.0)	NA	
4: Requiring oxygen	53 (58.2)	4 (8.0)	24 (48.0)	NA	
5: Requiring HFNC or NIV	12 (13.2)	0 (0.0)	2 (4.0)	NA	
6: Requiring IMV or ECMO	0 (0.0)	0 (0.0)	0 (0.0)	NA	
Delirium	1 (1.1)	0 (0.0)	1 (2.0)	NA	.62
Haloperidol	1 (100.0)	0(0.0)	1 (100.0)	NA	
mRS at discharge, mean (SD)	1.4 (0.9)	0.7 (0.7)	1.1 (1.0)	NA	< .001
**Laboratory findings, median (IQR)**					
Leukocytes (at admission)	6.1 (4.7, 7.5)	9.2 (8.0, 13.5)	11.2 (8.5, 14.7)	NA	< .001
Missing	0	23	0		
Leucocytes (peak)	8.0 (6.6, 11.6)	9.6 (8.0, 13.3)	12.6 (9.8, 17.0)	NA	.002
Missing	0	18	0		
Leukocytes (at discharge)	7.0 (5.6, 10.1)	7.8 (6.5, 10.3)	8.1 (6.5, 10.4)	NA	.31
Missing	0	18	1		
CRP (at admission)	62.0 (37.0, 126.0)	10.0 (4.0, 35.2)	137.0 (42.5, 259.2)	NA	< .001
Missing	0	28	0		
CRP (peak)	91.0 (51.5, 166.0)	17.0 (8.2, 48.0)	186.0 (73.8, 288.8)	NA	< .001
Missing	0	22	0		
CRP (at discharge)	19.0 (5.5, 42.0)	14.5 (7.3, 33.0)	46.0 (18.0, 65.0)	NA	< .001
Missing	0	20	1		

© 2023 Peinkhofer C et al. *JAMA Network Open*.

Creatinine (at admission)	77.0 (67.0, 91.5)	78.5 (69.8, 84.2)	78.5 (58.0, 94.5)	NA	.62
Missing	0	2	0	NA	
Creatinine (peak)	83.0 (70.5, 102.5)	83.0 (79.0, 94.0)	81.5 (64.2, 100.0)	NA	.35
Missing	0	1	0		
Creatinine (at discharge)	70.0 (61.0, 87.0)	78.0 (72.0, 89.5)	66.0 (56.0, 81.0)	NA	.02
Missing	0	6	0		
ALAT (at admission)	30.0 (21.0, 49.5)	29.0 (23.2, 55.2)	22.5 (17.2, 30.0)	NA	.02
Missing	1	40	4		
ALAT (peak)	63.5 (34.0, 113.0)	55.5 (28.5, 73.0)	31.0 (23.0, 73.0)	NA	.04
Missing	1	40	15		
ALAT (at discharge)	53.0 (28.0, 90.0)	36.0 (26.0, 59.5)	31.0 (19.5, 61.0)	NA	.17
Missing	3	42	23		
D-dimer (peak)	0.7 (0.4, 1.5)	0.4 (0.3, 3.1)	1.2 (0.8, 2.2)	NA	.50
Missing	25	45	13		
Creatin kinase (peak)	73.0 (41.5, 122.5)	1190.0 (597.0, 2357.5)	74.0 (72.5, 107.0)	NA	< .001
Missing	68	2	47		
LDH (peak)	328.5 (260.2, 436.5)	360.0 (249.0, 500.0)	213.5 (176.2, 267.2)	NA	< .001
Missing	5	41	2		
Days to follow-up, mean (SD)	640.6 (161.9)	542.1 (167.6)	727.9 (161.0)	NA	< .001
**Paraclinical characteristics**					
EEG	0(0.0)	0(0.0)	0(0.0)		
CT	1 (1.1)	0 (0.0)	3 (6.0)	NA	.07
Normal	1 (1.1)	0 (0.0)	1 (2.0)		.62
Leukoaraiosis	0 (0.0)	0 (0.0)	1 (2.0)		.24
Other	0 (0.0)	0 (0.0)	1 (2.0)		.24
MRI	3 (3.3)	0 (0.0)	0 (0.0)	NA	.19
Normal	3 (3.3)	0 (0.0)	0 (0.0)		.19
Nerve conduction studies	1 (1.1)	0 (0.0)	0 (0.0)	NA	.58
Normal	1 (1.1)	0 (0.0)	0 (0.0)		.58
Lumbar puncture	3 (3.3)	0 (0.0)	1 (2.0)	NA	.43
Pleocytosis	1 (1.1)	0 (0.0)	0 (0.0)		.58

Abbreviations: ALAT = Alanine aminotransferase; BMI = Body mass index; CRP = C Reactive Protein, COPD = Chronic obstructive pulmonary disease; CRP = C-reactive protein; ECMO = Extracorporeal Membrane Oxygenation; EEG = Electroencephalogram; FIRDA = Frontal Intermittent Rhythmic Delta Activity; HFNC= High Nasal Flow Cannula; ICU = Intensive Care Unit; IMV = Invasive Mechanical Ventilation; LDH= Lactate dehydrogenase; mRS = modified Rankin Scale; NIV = Non-Invasive Ventilation; OCD = Obsessive compulsive disorder; PTSD = Post-traumatic stress disorder; SAH = Subarachnoid Hemorrhage.

Data are presented as n (%), mean (SD), median (IQR).

a Linear model was used for comparison of means; log-linear model for comparison of medians; Pearson´s chi-squared for categorical variables

© 2023 Peinkhofer C et al. *JAMA Network Open*.

eTable 4. Baseline Characteristics of COVID-19 ICU and ICU Controls

	**Covid-19 ICU (n=29)**	**ICU controls (n=25)**	**p value[a]**
**Demographic**			
Age, mean (SD)	61.6 (12.3)	65.1 (13.0)	.32
Sex			.46
Female	11 (37.9)	12 (48.0)	
Male	11 (37.9)	12 (48.0)	
BMI, mean (SD)	31.2 (5.8)	26.4 (4.4)	.001
Smoking			.02
Current	0 (0.0)	6 (24.0)	
Never	13 (44.8)	9 (36.0)	
Previous	16 (55.2)	10 (40.0)	
Education			
Length, mean (SD)	12.6 (2.6)	12.8 (2.3)	.80
Grade			.73
Primary school	7 (24.1)	4 (16.0)	
Vocational training	10 (34.5)	7 (28.0)	
Short cycle	4 (13.8)	7 (28.0)	
Medium cycle	4 (13.8)	3 (12.0)	
Long cycle	4 (13.8)	4 (16.0)	
Covid-19 infection	29 (100.0)	5 (20.0)	< .001
Covid-19 vaccination	29 (100.0)	25 (100.0)	NA
**Previous medical history**			
Any comorbidity	23 (79.3)	20 (80.0)	.95
Hypertension	12 (41.4)	12 (48.0)	.63
Hyperlipidemia	6 (20.7)	7 (28.0)	.53
Diabetes Mellitus type II	5 (17.2)	4 (16.0)	.90
Malignancy	2 (6.9)	7 (28.0)	.04
Autoimmune disorder	2 (6.9)	2 (8.0)	.88
Asthma	5 (17.2)	0 (0.0)	.03
Other comorbidities	5 (17.2)	5 (20.0)	.80
COPD	0 (0.0)	3 (12.0)	.06
Interstitial lung disease	0 (0.0)	0 (0.0)	NA
Liver or pancreas	0 (0.0)	1 (4.0)	.28
Gastrointestinal	2 (6.9)	1 (4.0)	.64
Gynecology	1 (3.4)	1 (4.0)	.92
Urology	1 (3.4)	2 (8.0)	.47
Orthopedic	1 (3.4)	5 (20.0)	.05
Nephrology	1 (3.4)	0 (0.0)	.35
Major surgery in the last year	1 (3.4)	2 (8.0)	.47
Chronic infection	0(0.0)	0 (0.0)	NA
Other cardiac comorbidities	4 (13.8)	7 (28.0)	.20
**Previous neurological history**			
Any neurological comorbidity	5 (17.2)	5 (20.0)	.80
Traumatic brain injury	1 (3.4)	0 (0.0)	.35
Migraine	1 (3.4)	1 (4.0)	.92
Headache other	0 (0.0)	0 (0.0)	NA
Epilepsy	0 (0.0)	0 (0.0)	NA
Parkinson	1 (3.4)	1 (4.0)	.92

© 2023 Peinkhofer C et al. *JAMA Network Open.*

Movement disorders	0 (0.0)	0 (0.0)	NA
Multiple Sclerosis	0 (0.0)	0 (0.0)	NA
Neuromuscular	0 (0.0)	0 (0.0)	NA
Bone marrow injury	0 (0.0)	0 (0.0)	NA
Stroke	3 (10.3)	3 (12.0)	.85
Subarachnoid hemorrhage	1 (3.4)	1 (4.0)	.92
Benign brain tumor	0 (0.0)	0 (0.0)	NA
Neuroinfections	0 (0.0)	0 (0.0)	NA
Other neurological comorbidities	3 (10.3)	0 (0.0)	.10
**Previous psychiatric history**			
Any psychiatric comorbidity	10 (34.5)	8 (32.0)	.49
Depression	4 (13.8)	4 (16.0)	.82
Anxiety	2 (6.9)	2 (8.0)	.88
PTSD	0 (0.0)	0 (0.0)	NA
Stress	6 (20.7)	0 (0.0)	.39
Bipolar	0 (0.0)	0 (0.0)	NA
Schizophrenia	0(0.0)	0(0.0)	NA
OCD	0(0.0)	0(0.0)	NA
Alcohol abuse	0 (0.0)	3 (12.0)	.06
Drug abuse	1 (3.4)	0 (0.0)	.35
Eating disorder	1 (3.4)	0 (0.0)	.35
Other psychiatric comorbidities	1 (3.4)	1 (4.0)	.92
Relatives with psychiatric disorders			.25
Present	4 (13.8)	4 (16.0)	
Unknown	3 (10.3)	0 (0.0)	
Immunosuppression	8 (8.8)	0 (0.0)	.004
mRS before hospitalization (SD)	0.2 (0.5)	0.2 (0.6)	.66
Time from COVID-19 symptoms to hospitalization (SD)	8.0 (4.3)	NA	
Admission days (SD)	40.9 (24.4)	41.4 (23.3)	1.0
Severity scale (SD)	5.8 (0.4)	6.0 (0.2)	.07
Delirium	14 (48.3)	17 (68.0)	.14
Haloperidol	11 (78.6)	11 (64.7)	.40
Olanzapine	2 (14.3)	6 (37.5)	.15
Other	1 (7.1)	2 (12.5)	.63
Dialysis	8 (27.6)	7 (28.0)	.97
Inotropic agents	19 (65.5)	20 (80.0)	.24
Prone ventilation	19 (65.5)	0 (0.0)	<.001
ARDS	23 (79.3)	0 (0.0)	<.001
mRS at discharge, mean (SD)	3.1 (1.3)	3.1 (1.3)	.87
**Laboratory findings, median (IQR)**			
Leukocytes (at admission)	7.0 (5.0, 10.0)	11.0 (8.1, 14.1)	.06
Leucocytes (peak)	20.8 (15.0, 24.4)	19.0 (13.2, 24.3)	.49
Leukocytes (at discharge)	8.0 (6.2, 11.8)	8.0 (6.2, 9.3)	.26
CRP (at admission)	128.0 (60.0, 211.0)	11.0 (3.0, 86.0)	< .001
CRP (peak)	325.0 (286.0, 394.0)	310.0 (210.0, 345.0)	.09
CRP (at discharge)	8.0 (3.0, 16.0)	14.0 (12.0, 29.0)	.02
Creatinine (at admission)	92.0 (77.0, 105.0)	85.0 (65.0, 116.0)	.30
Creatinine (peak)	147.0 (108.0, 258.0)	163.0 (88.0, 211.0)	.92
Creatinine (at discharge)	77.0 (51.0, 87.0)	67.0 (59.0, 85.0)	.76
ALAT (at admission)	51.0 (34.0, 74.0)	34.5 (17.8, 65.5)	.46
ALAT (peak)	120.0 (89.0, 211.0)	197.0 (84.0, 324.0)	.19
ALAT (at discharge)	33.0 (28.0, 57.0)	53.0 (34.0, 79.0)	.25
D-dimer (peak)	133.0 (108.2, 467.2)	312.0 (185.5, 887.0)	.003

© 2023 Peinkhofer C et al. *JAMA Network Open.*

Creatin kinase (peak)	73.0 (41.5, 122.5)	1190.0 (597.0, 2357.5)	0.237
LDH (peak)	599.0 (492.0, 805.0)	462.0 (401.0, 783.0)	0.212
Days to follow-up, mean (SD)	572.0 (400.0, 614.0)	604.0 (570.0, 690.0)	0.002
**Paraclinical characteristics**			
EEG	5 (17.2)	3 (12.0)	.59
Normal	0 (0.0)	1 (4.0)	.28
FIRDA	2 (6.9)	0 (0.0)	.18
Sharp waves	1 (3.4)	0 (0.0)	.35
Encephalopathy	2 (6.9)	2 (8.0)	.88
CT	10 (34.5)	9 (36.0)	.91
Normal	5 (17.2)	6 (24.0)	.54
Old stroke	2 (6.9)	1 (4.0)	.64
Leukoaraiosis	0 (0.0)	1 (4.0)	.28
New hemorrhagic stroke	1 (3.4)	0 (0.0)	.35
SAH	2 (6.9)	0 (0.0)	.18
Other	0 (0.0)	1 (4.0)	.28
MRI	5 (17.2)	3 (12.0)	.59
Normal	0 (0.0)	1 (4.0)	.28
Old stroke	1 (3.4)	1 (4.0)	.92
Leukoaraiosis	1 (3.4)	0 (0.0)	.35
New hemorrhagic stroke	1 (3.4)	0 (0.0)	.35
Hydrocephalus	1 (3.4)	0 (0.0)	.35
SAH	1 (3.4)	0 (0.0)	.35
Other	1 (3.4)	1 (4.0)	.92
Nerve conduction studies	2 (6.9)	1 (4.0)	.64
Normal	1 (3.4)	0 (0.0)	.35
Critical illness neuropathy	0 (0.0)	1 (4.0)	.28
Critical illness myopathy	1 (3.4)	0 (0.0)	.35
Lumbar puncture	3 (10.3)	3 (12.0)	.85
Pleocytosis	0 (0.0)	1 (4.0)	.28

Abbreviations: ALAT = Alanine aminotransferase; BMI = Body mass index; CRP = C Reactive Protein, COPD = Chronic obstructive pulmonary disease; CRP = C-reactive protein; ECMO = Extracorporeal membrane oxygenation; EEG = Electroencephalogram; FIRDA = Frontal intermittent rhythmic delta activity; HFNC= High nasal flow cannula; ICU = Intensive care unit; IMV = Invasive mechanical ventilation; LDH= Lactate dehydrogenase; mRS = modified Rankin Scale; NIV = Non-invasive ventilation; OCD = Obsessive compulsive disorder; PTSD = Post-traumatic stress disorder; SAH = Subarachnoid hemorrhage.
Data are presented as n (%), mean (SD), median (IQR).
a Linear model was used for comparison of means; log-linear model for comparison of medians; Pearson´s chi-squared for categorical variables

© 2023 Peinkhofer C et al. *JAMA Network Open.*

eTable 5. Cognitive, Neurological, and Psychiatric Outcomes Compared Between COVID-19 Patients, All Hospitalized Individuals and Healthy Controls

	Group	N	Geometric Mean (95% CI)	Estimated Mean (95% CI)	RMD (95%CI)[a]	p value[b]	Het p[c]
**Primary outcomes**							
SCIP	COVID-19	113	58.6 (56.7 - 60.6)	59.0 (56.9 - 61.2)			
SCIP	Hospitalized	124	61.5 (59.5 - 63.5)	62.3 (60.3 - 64.5)	0.95 (0.90 - 1.00)	.03	
SCIP	Healthy	100	70.5 (68.1 - 73.1)	68.8 (66.2 - 71.5)	0.86 (0.81 - 0.91)	<.001	<.001
MoCA	COVID-19	120	26.5 (26.0 - 26.9)	26.5 (26.0 - 27.0)			
MoCA	Hospitalized	125	27.3 (26.9 - 27.7)	27.4 (27.0 - 27.8)	0.84 (0.74 - 0.96)	.01	
MoCA	Healthy	100	28.4 (28.0 - 28.7)	28.2 (27.8 - 28.6)	0.69 (0.59 - 0.79)	<.001	<.001
**Secondary outcomes**							
Hamilton Anxiety	COVID-19	120	4.0 (3.4 - 4.7)	3.7 (3.1 - 4.4)			
Hamilton Anxiety	Hospitalized	125	2.6 (2.2 - 3.1)	2.7 (2.3 - 3.2)	1.35 (1.06 - 1.73)	.01	
Hamilton Anxiety	Healthy	100	2.3 (2.0 - 2.8)	2.5 (2.0 - 3.0)	1.49 (1.14 - 1.94)	.003	.007
Hamilton Depression	COVID-19	120	4.1 (3.5 - 4.8)	3.8 (3.2 - 4.5)			
Hamilton Depression	Hospitalized	125	3.1 (2.7 - 3.7)	3.2 (2.7 - 3.8)	1.20 (0.94 - 1.51)	.134	
Hamilton Depression	Healthy	100	2.3 (2.0 - 2.7)	2.5 (2.0 - 3.0)	1.56 (1.21 - 2.03)	.001	.004
Neurological evaluation scale	COVID-19	119	7.9 (7.0 - 9.0)	7.8 (6.8 - 8.9)			
Neurological evaluation scale	Hospitalized	125	7.9 (7.0 - 9.0)	7.4 (6.5 - 8.4)	1.05 (0.87 - 1.28)	.59	
Neurological evaluation scale	Healthy	100	5.1 (4.5 - 5.9)	5.7 (4.9 - 6.6)	1.37 (1.11 - 1.69)	.004	.008
Trail A	COVID-19	119	34.9 (32.7 - 37.2)	36.1 (33.7 - 38.7)			
Trail A	Hospitalized	125	33.6 (31.5 - 35.7)	31.3 (29.3 - 33.5)	1.15 (1.04 - 1.27)	.005	
Trail A	Healthy	100	29.0 (27.0 - 31.1)	30.3 (28.1 - 32.7)	1.19 (1.07 - 1.33)	.002	.003
Trail B	COVID-19	118	87.8 (81.0 - 95.1)	90.7 (83.3 - 98.9)			
Trail B	Hospitalized	125	84.7 (78.4 - 91.6)	79.7 (73.5 - 86.5)	1.14 (1.01 - 1.29)	.04	
Trail B	Healthy	100	66.2 (60.7 - 72.2)	68.7 (62.5 - 75.5)	1.32 (1.16 - 1.51)	<.001	<.001

Abbreviations: Het p = Effect heterogeneity p values ; MoCA= Montreal cognitive assessment; RMD = Relative mean difference; SCIP = Screening for cognitive impairment in psychiatry

Data are presented as number of participants, geometric and estimated means with 95% CI.

a Relative mean difference for MoCA scores refers to mean difference of "32 – MoCA score" and for Hamilton Anxiety, Hamilton Depression and the Neurological evaluation scale to "total score +1"

b Models are adjusted for sex, age, BMI, education grade, alcohol abuse, smoking and ICU admission

c Effect heterogeneity p-values are based on the likelihood ratio statistic

© 2023 Peinkhofer C et al. *JAMA Network Open.*

eTable 6. Cognitive, Neurological, and Psychiatric Outcomes Compared Between Non-ICU COVID-19 Patients and the Other Non-ICU Groups in Models Adjusted for Age and Sex (A) and Fully Adjusted (B)

A	Group	N	Geometric Mean (95% CI)	Estimated Mean (95% CI)	RMD (95%CI)[a]	p value[b]	Het p[c]
**Primary outcomes**							
SCIP	COVID-19	88	58.5 (56.2 - 60.9)	57.6 (55.3 - 60.0)			
SCIP	AMI	50	61.6 (58.3 - 65.0)	61.6 (58.3 - 65.0)	0.94 (0.87 - 1.00)	.05	
SCIP	Pneumonia	49	61.8 (58.6 - 65.3)	64.2 (60.8 - 67.9)	0.90 (0.84 - 0.96)	.002	
SCIP	Healthy	100	70.5 (67.9 - 73.3)	70.2 (67.6 - 72.9)	0.82 (0.78 - 0.87)	<.001	<.001
MoCA	COVID-19	91	26.5 (25.9 - 27.0)	26.3 (25.7 - 26.9)			
MoCA	AMI	50	27.2 (26.5 - 27.8)	27.2 (26.5 - 27.8)	0.85 (0.71 - 1.01)	.06	
MoCA	Pneumonia	50	27.2 (26.5 - 27.8)	27.4 (26.7 - 28.0)	0.81 (0.68 - 0.97)	.02	
MoCA	Healthy	100	28.4 (28.0 - 28.7)	28.4 (28.0 - 28.7)	0.64 (0.56 - 0.74)	<.001	<.001
**Secondary outcomes**							
Hamilton Anxiety	COVID-19	91	3.8 (3.2 - 4.6)	3.8 (3.1 - 4.5)			
Hamilton Anxiety	AMI	50	2.6 (2.0 - 3.3)	2.7 (2.1 - 3.5)	1.40 (1.02 - 1.91)	.04	
Hamilton Anxiety	Pneumonia	50	2.3 (1.8 - 2.9)	2.3 (1.8 - 3.0)	1.62 (1.18 - 2.23)	.003	
Hamilton Anxiety	Healthy	100	2.3 (2.0 - 2.8)	2.3 (1.9 - 2.7)	1.67 (1.29 - 2.16)	<.001	.001
Hamilton Depression	COVID-19	91	3.9 (3.2 - 4.7)	3.9 (3.2 - 4.7)			
Hamilton Depression	AMI	50	3.2 (2.5 - 4.0)	3.3 (2.6 - 4.2)	1.18 (0.87 - 1.60)	.29	
Hamilton Depression	Pneumonia	50	2.7 (2.1 - 3.5)	2.7 (2.1 - 3.5)	1.42 (1.04 - 1.94)	.03	
Hamilton Depression	Healthy	100	2.3 (1.9 - 2.7)	2.3 (1.9 - 2.7)	1.73 (1.34 - 2.22)	<.001	<.001
Neurological evaluation scale	COVID-19	90	7.6 (6.5 - 8.8)	8.3 (7.1 - 9.7)			
Neurological evaluation scale	AMI	50	7.1 (5.8 - 8.8)	7.2 (5.8 - 8.9)	1.16 (0.89 - 1.50)	.28	
Neurological evaluation scale	Pneumonia	50	7.8 (6.3 - 9.6)	6.4 (5.2 - 7.9)	1.30 (0.99 - 1.69)	.06	
Neurological evaluation scale	Healthy	100	5.1 (4.4 - 5.9)	5.2 (4.5 - 6.0)	1.60 (1.29 - 1.99)	<.001	<.001
Trail A	COVID-19	90	33.4 (31.1 - 35.9)	35.3 (32.8 - 37.9)			
Trail A	AMI	50	31.1 (28.2 - 34.2)	31.4 (28.5 - 34.6)	1.12 (0.99 - 1.27)	.06	
Trail A	Pneumonia	50	34.3 (31.1 - 37.8)	30.7 (27.8 - 33.8)	1.15 (1.02 - 1.30)	.03	
Trail A	Healthy	100	29.0 (27.0 - 31.0)	29.0 (27.1 - 31.1)	1.22 (1.10 - 1.34)	<.001	.002
Trail B	COVID-19	89	85.5 (78.0 - 93.7)	90.6 (82.6 - 99.4)			
Trail B	AMI	50	77.1 (68.3 - 87.2)	78.2 (69.2 - 88.5)	1.16 (0.99 - 1.35)	.06	
Trail B	Pneumonia	50	89.4 (79.1 - 101.1)	79.1 (69.9 - 89.6)	1.15 (0.98 - 1.34)	.09	
Trail B	Healthy	100	66.2 (60.7 - 72.1)	66.3 (60.8 - 72.3)	1.37 (1.20 - 1.55)	<.001	<.001

Abbreviations: AMI = Acute myocardial infarction; Het p = Effect heterogeneity p values; MoCA= Montreal cognitive assessment; RMD =Relative mean difference; SCIP = Screening for cognitive impairment in psychiatry
Data are presented as number of participants, geometric and estimated means with 95% CI.
a Relative mean difference for MoCA scores refers to mean difference of "32 – MoCA score" and for Hamilton Anxiety, Hamilton Depression and the Neurological evaluation scale to "total score +1"
b Models are adjusted only for age and sex
c Effect heterogeneity p-values are based on the likelihood ratio statistic

© 2023 Peinkhofer C et al. *JAMA Network Open.*

B	Group	N	Geometric Mean (95% CI)	Estimated Mean (95% CI)	RMD (95%CI)[a]	p value[b]	Het p[c]
**Primary outcomes**							
SCIP	COVID-19	88	58.5 (56.0 - 61.2)	58.2 (55.6 - 61.0)			
SCIP	AMI	50	61.6 (58.0 - 65.3)	60.9 (57.3 - 64.8)	0.96 (0.89 - 1.03)	.25	
SCIP	Pneumonia	49	61.8 (58.3 - 65.6)	63.0 (58.9 - 67.3)	0.92 (0.85 - 1.01)	.07	.17
MoCA	COVID-19	91	26.5 (25.9 - 27.0)	26.4 (25.8 - 27.0)			
MoCA	AMI	50	27.2 (26.5 - 27.9)	27.2 (26.4 - 27.9)	0.86 (0.72 - 1.03)	.11	
MoCA	Pneumonia	50	27.2 (26.4 - 27.8)	27.3 (26.5 - 28.0)	0.85 (0.69 - 1.04)	.11	.15
**Secondary outcomes**							
Hamilton Anxiety	COVID-19	91	3.8 (3.1 - 4.6)	3.5 (2.8 - 4.2)			
Hamilton Anxiety	AMI	50	2.6 (2.0 - 3.3)	2.7 (2.1 - 3.5)	1.29 (0.93 - 1.80)	.13	
Hamilton Anxiety	Pneumonia	50	2.3 (1.8 - 2.9)	2.6 (1.9 - 3.4)	1.34 (0.93 - 1.94)	.12	.17
Hamilton Depression	COVID-19	91	3.9 (3.2 - 4.7)	3.6 (2.9 - 4.3)			
Hamilton Depression	AMI	50	3.2 (2.5 - 4.0)	3.3 (2.6 - 4.3)	1.07 (0.77 - 1.48)	.68	
Hamilton Depression	Pneumonia	50	2.7 (2.1 - 3.5)	3.0 (2.3 - 4.0)	1.17 (0.82 - 1.68)	.39	.68
Neurological evaluation scale	COVID-19	90	7.6 (6.5 - 8.9)	8.1 (6.8 - 9.5)			
Neurological evaluation scale	AMI	50	7.1 (5.8 - 8.8)	7.5 (6.0 - 9.3)	1.08 (0.82 - 1.42)	.58	
Neurological evaluation scale	Pneumonia	50	7.8 (6.3 - 9.6)	6.7 (5.3 - 8.4)	1.21 (0.90 - 1.64)	.21	.45
Trail A	COVID-19	90	33.4 (31.0 - 36.0)	35.0 (32.4 - 37.9)			
Trail A	AMI	50	31.1 (28.1 - 34.3)	31.9 (28.8 - 35.4)	1.10 (0.96 - 1.25)	.16	
Trail A	Pneumonia	50	34.3 (31.0 - 37.9)	30.7 (27.4 - 34.4)	1.14 (0.99 - 1.32)	.07	.14
Trail B	COVID-19	89	85.5 (78.1 - 93.5)	89.7 (81.6 - 98.5)			
Trail B	AMI	50	77.1 (68.4 - 87.0)	80.5 (71.1 - 91.1)	1.11 (0.95 - 1.30)	.17	
Trail B	Pneumonia	50	89.4 (79.3 - 100.8)	78.7 (68.9 - 90.1)	1.14 (0.96 - 1.35)	.14	.22

Abbreviations: AMI = Acute myocardial infarction; Het p = Effect heterogeneity p values ; MoCA= Montreal cognitive assessment; RMD = Relative mean difference; SCIP = Screening for cognitive impairment in psychiatry
Data are presented as number of participants, geometric and estimated means with 95% CI.
a Relative mean difference for MoCA scores refers to mean difference of "32 – MoCA score" and for Hamilton Anxiety, Hamilton Depression and the Neurological evaluation scale to "total score +1"
b Models are adjusted for sex, age, admission length and time from hospitalization to follow-up
c Effect heterogeneity p-values are based on the likelihood ratio statistic

© 2023 Peinkhofer C et al. *JAMA Network Open.*

eTable 7. Cognitive, Neurological, and Psychiatric Outcomes Compared Between ICU COVID-19 and ICU Controls in Models Adjusted for Age and Sex (A) and Fully Adjusted (B)

A	Group	N	Geometric Mean (95% CI)	Estimated Mean (95% CI)	RMD (95%CI)[a]	p value[b]	Het p[c]
**Primary outcomes**							
SCIP	COVID-19	29	59.1 (54.3 - 64.2)	58.8 (54.0 - 63.9)			
SCIP	ICU	25	60.6 (55.7 - 65.9)	60.9 (55.9 - 66.2)	0.97 (0.86 - 1.09)	.56	.56
MoCA	COVID-19	29	26.4 (25.2 - 27.3)	26.4 (25.2 - 27.3)			
MoCA	ICU	25	27.7 (26.7 - 28.5)	27.7 (26.7 - 28.5)	0.76 (0.58 - 1.01)	.06	.06
**Secondary outcomes**							
Hamilton Anxiety	COVID-19	29	4.9 (3.4 - 7.1)	4.8 (3.3 - 7.0)			
Hamilton Anxiety	ICU	25	3.6 (2.4 - 5.3)	3.7 (2.5 - 5.5)	1.30 (0.75 - 2.27)	.35	.35
Hamilton Depression	COVID-19	29	5.0 (3.4 - 7.2)	5.0 (3.4 - 7.2)			
Hamilton Depression	ICU	25	4.2 (2.8 - 6.2)	4.2 (2.8 - 6.3)	1.18 (0.68 - 2.04)	.55	.55
Neurological evaluation scale	COVID-19	29	9.0 (6.9 - 11.8)	9.6 (7.3 - 12.6)			
Neurological evaluation scale	ICU	25	10.1 (7.6 - 13.6)	9.4 (7.0 - 12.7)	1.02 (0.68 - 1.53)	.94	.94
Trail A	COVID-19	29	39.8 (33.8 - 46.9)	40.6 (34.5 - 47.9)			
Trail A	ICU	25	37.5 (31.5 - 44.8)	36.7 (30.7 - 43.8)	1.11 (0.87 - 1.41)	.41	.41
Trail B	COVID-19	29	95.2 (78.7 - 115.1)	99.8 (82.4 - 120.9)			
Trail B	ICU	25	91.8 (74.8 - 112.7)	86.9 (70.7 - 106.9)	1.15 (0.86 - 1.52)	.33	.33

Abbreviations: Het p = Effect heterogeneity p values; ICU = Intensive care unit; MoCA= Montreal cognitive assessment; RMD = Relative mean difference; SCIP = Screening for cognitive impairment in psychiatry
Data are presented as number of participants, geometric and estimated means with 95% CI.
a Relative mean difference for MoCA scores refers to mean difference of "32 – MoCA score" and for Hamilton Anxiety, Hamilton Depression and the Neurological evaluation scale to "total score +1"
b Models are adjusted for sex and age.
c Effect heterogeneity p-values are based on the likelihood ratio statistic

B	Group	N	Geometric Mean (95% CI)	Estimated Mean (95% CI)	RMD (95%CI)[a]	p value[b]	Het p[c]
**Primary outcomes**							
SCIP	COVID-19	29	59.1 (54.2 - 64.4)	58.7 (53.5 - 64.3)			
SCIP	ICU	25	60.6 (55.6 - 66.0)	61.0 (55.6 - 66.8)	0.96 (0.84 - 1.10)	.58	.58
MoCA	COVID-19	29	26.4 (25.2 - 27.4)	26.5 (25.3 - 27.5)			
MoCA	ICU	25	27.7 (26.7 - 28.5)	27.5 (26.5 - 28.4)	0.81 (0.60 - 1.11)	.19	.19
**Secondary outcomes**							
Hamilton Anxiety	COVID-19	29	4.9 (3.4 - 7.1)	4.7 (3.1 - 6.9)			
Hamilton Anxiety	ICU	25	3.6 (2.4 - 5.3)	3.8 (2.5 - 5.8)	1.23 (0.67 - 2.27)	.50	.50
Hamilton Depression	COVID-19	29	5.0 (3.4 - 7.2)	4.9 (3.3 - 7.2)			
Hamilton Depression	ICU	25	4.2 (2.8 - 6.2)	4.3 (2.8 - 6.5)	1.15 (0.63 - 2.10)	.65	.65
Neurological evaluation scale	COVID-19	29	9.0 (6.8 - 11.9)	9.5 (7.1 - 12.8)			
Neurological evaluation scale	ICU	25	10.1 (7.5 - 13.7)	9.5 (6.9 - 13.1)	1.00 (0.63 - 1.58)	.99	0.99
Trail A	COVID-19	29	39.8 (33.8 - 47.0)	41.3 (34.7 - 49.2)			
Trail A	ICU	25	37.5 (31.4 - 44.8)	35.9 (29.7 - 43.5)	1.15 (0.88 - 1.51)	.31	.31
Trail B	COVID-19	29	95.2 (78.5 - 115.5)	100.7 (82.1 - 123.6)			
Trail B	ICU	25	91.8 (74.6 - 113.1)	86.0 (68.8 - 107.4)	1.17 (0.85 - 1.61)	.32	.32

© 2023 Peinkhofer C et al. *JAMA Network Open.*

Abbreviations: Het p = Effect heterogeneity p values; ICU = Intensive care unit; MoCA= Montreal cognitive assessment; RMD = Relative Mean difference; SCIP = Screening for cognitive impairment in psychiatry
Data are presented as number of participants, geometric and estimated means with 95% CI.
a Relative mean difference for MoCA scores refers to mean difference of "32 – MoCA score" and for Hamilton Anxiety, Hamilton Depression and the Neurological evaluation scale to "total score +1"
b Models are adjusted for sex, age, admission length and time from hospitalization to follow-up
c Effect heterogeneity p-values are based on the likelihood ratio statistic

© 2023 Peinkhofer C et al. *JAMA Network Open*.

4a. Exploratory outcomes tables

eTable 8. Self-Reported Neuropsychiatric Symptoms in COVID-19 Patients and Hospitalized Controls at the 18 Months Investigation

**Subjective neuropsychiatric symptoms**	**COVID-19 (n=120)**	**Hospitalized controls (n = 125)**	**OR or COR[*] (95% CI)[a]**	**p value[b]**
Anosmia ever	65 (54.2)	13 (10.4)	9.85 (4.97 - 19.52)	<.001
Anosmia at follow-up			0.17 (0.07 - 0.41) [*]	<.001
No	90 (75.0)	117 (93.6)		
Partial	25 (20.8)	8 (6.4)		
Total	5 (4.2)	0 (0.0)		
Olfactory hallucination	21 (17.5)	3 (2.4)	7.72 (2.21 - 26.93)	.001
Dysgeusia ever	68 (56.7)	24 (19.2)	5.54 (3.09 - 9.95)	<.001
Dysgeusia at follow-up			0.39 (0.18 - 0.83) [*]	.01
No	96 (80.0)	112 (89.6)		
Partial	21 (17.5)	12 (9.6)		
Total	3 (2.5)	1 (0.8)		
Gustatory hallucination	6 (5.0)	0 (0.0)	NA	NA
Vision	23 (19.2)	24 (19.2)	0.92 (0.48 - 1.76)	.79
´Paresthesia[*]			1.08 (0.60 - 1.95) [*]	.79
No	87 (72.5)	95 (76.0)		
Mild	33 (27.5)	33 (26.4)		
Moderate	30 (25.0)	22 (17.6)		
Severe	9 (7.5)	1 (0.8)		
New headache	12 (10.0)	3 (2.4)	NA	NA
Worsening of previous headache	22 (18.3)	8 (6.4)	2.75 (1.15 - 6.59)	.02
Dizziness			0.81 (0.40 - 1.64) [*]	.59
No	103 (85.8)	103 (82.4)		
Mild	11 (9.2)	15 (12.0)		
Moderate	5 (4.2)	6 (4.8)		
Severe	1 (0.8)	1 (0.8)		
Tinnitus			2.09 (1.00 - 4.37) [*]	.05
No	96 (80.0)	112 (89.6)		
Mild	18 (15.0)	9 (7.2)		
Moderate	3 (2.5)	2 (1.6)		
Severe	3 (2.5)	2 (1.6)		
Pain			1.41 (0.77 - 2.58) [*]	.27
No	87 (72.5)	100 (80.0)		
Mild	22 (18.3)	15 (12.0)		
Moderate	9 (7.5)	7 (5.6)		
Severe	2 (1.7)	3 (2.4)		
Sleep	52 (43.3)	39 (31.2)	1.60 (0.94 - 2.74)	.08
Short memory problems			1.91 (1.18 - 3.10) [*]	.009
No	48 (40.0)	69 (55.2)		
Mild	33 (27.5)	33 (26.4)		
Moderate	30 (25.0)	22 (17.6)		

© 2023 Peinkhofer C et al. *JAMA Network Open.*

Severe	9 (7.5)	1 (0.8)		
Long memory problems			1.24 (0.56 - 2.75) *	0.60
No	104 (86.7)	112 (89.6)		
Mild	10 (8.3)	11 (8.8)		
Moderate	5 (4.2)	2 (1.6)		
Severe	1 (0.8)	0 (0.0)		
Concentration			1.94 (1.11 - 3.38) *	.02
No	71 (59.2)	93 (74.4)		
Mild	17 (14.2)	21 (16.8)		
Moderate	24 (20.0)	9 (7.2)		
Severe	8 (6.7)	2 (1.6)		
Speech impairment			1.34 (0.80 - 2.24) *	.27
No	68 (56.7)	77 (61.6)		
Sometimes	30 (25.0)	33 (26.4)		
Often	20 (16.7)	15 (12.0)		
Daily	2 (1.7)	0 (0.0)		
Irritability	10 (8.3)	6 (4.8)	1.23 (0.39 - 3.84) *	.72
Emotional lability	11 (9.2)	4 (3.2)	NA	NA
Flashbacks ever			1.07 (0.55 - 2.08) *	.85
No	95 (79.2)	103 (82.4)		
Sometimes	13 (10.8)	16 (12.8)		
Often	12 (10.0)	6 (4.8)		
Social isolation			1.46 (0.81 - 2.61) *	.21
No	84 (70.0)	97 (77.6)		
Mild	19 (15.8)	12 (9.6)		
Moderate	14 (11.7)	13 (10.4)		
Severe	3 (2.5)	3 (2.4)		
Psychological help			0.66 (0.30 - 1.46) *	.30
No	103 (85.8)	107 (85.6)		
Short (<1 year)	8 (6.7)	12 (9.6)		
Long (>1 year)	9 (7.5)	6 (4.8)		

Abbreviations: OR = Odds ratio; COR = Cumulative odds ratio
Data are n(%). Comparison in groups with ≤ 5 are not reported.
a OR presented with 95% CI from logistic regression models for binary outcomes (yes/no) and COR (no/mild/moderate/severe) with 95% CI from proportional odds models for ordinal outcomes. Both models are adjusted for age and sex. Confidence intervals are based on the Wald statistic
b P-values are based on the Wald statistic and are significant if <0.001 after multiplicity adjustment
*Cumulative odds ratio for ordinal outcomes

© 2023 Peinkhofer C et al. *JAMA Network Open.*

eTable 9. Neurological Examination Findings in COVID-19 Patients, Hospitalized Controls and Healthy Controls at the 18 Months Investigation

	COVID-19 (n=119)[a]	Hospitalized controls (n = 125)	Healthy controls (n=100)	COVID-19 vs hospitalized OR (95% CI)[b]	p value[c]	COVID-19 vs healthy OR (95% CI)[b]	p value[c]	Het p[d]
**Neurological exam**								
Motor deficit	2 (1.7)	2 (1.6)	0 (0.0)	··	··	··	··	··
Gait deficit	3 (2.5)	15 (12.0)	0 (0.0)	··	··	··	··	··
Strength deficit	24 (20.2)	21 (16.8)	9 (9.0)	0.70 (0.36 - 1.36)	.29	0.34 (0.15 - 0.78)	.01	.08
Sensory deficit	9 (7.6)	9 (7.2)	4 (4.0)	0.96 (0.36 - 2.55)	.93	··	··	··
Coordination deficit	8 (6.7)	9 (7.2)	6 (6.0)	0.92 (0.34 - 2.52)	.88	0.87 (0.28 - 2.65)	.80	.91
Reflex abnormality	32 (26.9)	33 (26.4)	14 (14.0)	0.82 (0.46 - 1.48)	.52	0.40 (0.19 - 0.81)	.01	.04
Olfactory nerve abnormality [e]	46 (38.7)	36 (28.8)	16 (16.0)	0.39 (0.22 - 0.70)	.001	0.20 (0.10 - 0.40)	<.001	.05
Other cranial nerves abnormality	7 (5.9)	5 (4.0)	1 (1.0)	··	··	··	··	··
Presence of primitive reflexes	17 (14.3)	8 (6.4)	4 (4.0)	2.82 (1.15 - 6.91)	.02	··	··	··
Sniff test[f]				0.42 (0.24 - 0.74)[f]	.002	0.23 (0.12 - 0.44)[f]	<.001	.06
0/4	2 (2.0)	3 (2.4)	0 (0.0)					
1/4	2 (2.0)	4 (3.2)	0 (0.0)					
2/4	8 (8.2)	9 (7.2)	2 (2.0)					
3/4	36 (36.7)	19 (15.2)	16 (16.0)					
4/4	50 (51.0)	90 (72.0)	82 (82.0)					

Abbreviations: OR = Odds ratio; Het p = Effect heterogeneity p values
Data are n(%).Comparison in groups with ≤ 5 are not reported.

a Missing data from 1 COVID-19 patient
b OR presented with 95% CI from logistic regression models adjusted for age and sex. Confidence intervals are based on the Wald statistic
c P-values are based on the Wald statistic and are significant if <0.001 after multiplicity adjustment
d Heterogeneity p-values were based on the likelihood ratio statistic
e Missing data from 22 COVID-19 patients
f Ordinal outcome, results are not odds ratio but cumulative odds ratio
·· = N/A

© 2023 Peinkhofer C et al. *JAMA Network Open.*

eTable 10. New Onset Psychiatric Disorders Assessed With the MINI Interview in COVID-19 Patients, Hospitalized Controls and Healthy Controls at the 18 Months Investigation

	COVID-19 (n=120)	Hospitalized controls (n = 125)	Healthy controls (n=100)	COVID-19 vs hospitalized OR (95% CI)[a]	p value[b]	COVID-19 vs healthy OR (95% CI)[a]	p value[b]	Het p[c]
Any new psychiatric diagnoses	41 (34.2)	24 (19.2)	5(5.0)	2.05 (1.12 - 3.75)	.02	11.24 (4.17 – 30.31)	<.001	<.001
Major depression	24 (20.0)	12 (9.6)	2 (2.0)	2.37 (1.09 - 5.18)	.03	NA	NA	NA
Suicidality	11 (9.2)	3 (2.4)	0 (0.0)	NA	NA	NA	NA	NA
Generalized anxiety	10 (8.3)	5 (4.0)	3 (3.0)	1.75 (0.56 - 5.46)	.33	NA	NA	NA
Panic disorder	8 (6.7)	7 (5.6)	0 (0.0)	1.01 (0.34 - 3.01)	.99	NA	NA	NA
Panic disorders with agoraphobia	2 (1.7)	1 (0.8)	0 (0.0)	NA	NA	NA	NA	NA
Agoraphobia	4 (3.3)	4 (3.2)	0 (0.0)	NA	NA	NA	NA	NA
Social anxiety disorder	3 (2.5)	5 (4.0)	0 (0.0)	NA	NA	NA	NA	NA
Hypomania	2 (1.7)	1 (0.8)	0 (0.0)	NA	NA	NA	NA	NA
Mania	0 (0.0)	0 (0.0)	0 (0.0)	NA	NA	NA	NA	NA
Obsessive compulsive disorder	0 (0.0)	1 (0.8)	0 (0.0)	NA	NA	NA	NA	NA
Post-traumatic stress disorder	6 (5.0)	6 (4.8)	0 (0.0)	0.53 (0.14 - 1.95)	0.34	NA	NA	NA
Psychotic disorder	1 (0.8)	0 (0.0)	0 (0.0)	NA	NA	NA	NA	NA

Abbreviations: OR = Odds ratio; Het p = Effect heterogeneity p values
Data are n(%).Comparison in groups with ≤ 5 are not reported.
a OR presented with 95% CI from logistic regression models adjusted for age and sex. Confidence intervals are based on the Wald statistic
b P-values are based on the Wald statistic and are significant if <0.001 after multiplicity adjustment
c Heterogeneity p-values were based on the likelihood ratio statistic

© 2023 Peinkhofer C et al. *JAMA Network Open.*

eTable 11. Fatigue Assessment Scale Scores Compared Between COVID-19 Patients, Hospitalized Controls and Healthy Controls

	Group	N	Geometric Mean (95% CI)	Estimated Mean (95% CI)	RMD (95%CI)	p value[a]	Het p[b]
FAS	COVID-19	108	22.0 (20.4 - 23.8)	20.8 (19.2 - 22.6)			
FAS	Hospitalized	125	17.7 (16.5 - 19.0)	17.9 (16.6 - 19.3)	1.16 (1.04 - 1.30)	.01	
FAS	Healthy	100	13.4 (12.4 - 14.5)	14.1 (12.9 - 15.4)	1.48 (1.30 - 1.68)	<.001	<.001

Abbreviations: FAS = Fatigue assessment scale; Het p = Effect heterogeneity p values; RMD = Relative mean difference
Data are presented as number of participants, geometric and estimated means with 95% CI.
a Models are adjusted for age and sex
b Effect heterogeneity p-values are based on the likelihood ratio statistic

© 2023 Peinkhofer C et al. *JAMA Network Open.*

eTable 12. Changes in Mean MoCA Score Over Time in COVID-19 Individuals

	0 months (n = 16) *	6 months (n=56) **	18 months (n=120)	0 vs 18 months RMD (95%CI)[a]	p value[b]	6 vs 18 months RMD (95%CI)[a]	p value[b]
MoCA scores	21.7 (18.7 - 23.9)	27.4 (26.7 - 28.0)	26.5 (25.9 - 27.0)	0.54 (0.42 - 0.69)	<.001	1.21 (1.04 - 1.40)	.01

Abbreviations: MoCA = Montreal cognitive assessment; RMD= Relative mean difference
a Relative mean difference for MoCA scores refers to mean difference of "32 – MoCA score"
b Models are adjusted for sex and age
*Results are previously published [1]
** Results are previously published [2]

© 2023 Peinkhofer C et al. *JAMA Network Open.*

eTable 13. Changes in Frequency of Neurological Findings, Psychiatric Diagnoses and Subjective Symptoms in COVID-19 Individuals Between 6- and 18-Months Follow-up Visits

	COVID-19 (n=56)			
	**6 months**	**18 months**	**OR (95%CI)[a]**	**p value[b]**
**Psychiatric and neurologic**				
Any new psychiatric diagnosis	10 (17.9)	18 (32.1)	3.00 (0.97 - 9.28)	.04
Any neurological abnormality	14 (25.0)	29 (51.8)	4.75 (1.62 - 13.96)	.001
**Subjective neuropsychiatric symptoms**				
Memory	24 (42.9)	36 (64.3)	4.98 (1.45 - 17.10)	.003
Concentration	27 (48.2)	28 (50.0)	1.24 (0.34 - 4.49)	.74
Speech impairment	2 (3.6)	26 (46.4)	··	··
Irritability	17 (30.4)	6 (10.7)	0.15 (0.04 - 0.68)	.003
Emotional lability	16 (28.6)	7 (12.5)	0.25 (0.07 - 0.88)	.02
Flashbacks, ever	4 (7.1)	15 (26.8)	··	··
**Subjective neurological symptoms**				
Anosmia	13 (23.2)	13 (23.2)	1.20 (0.37 - 3.89)	.77
Dysgeusia	13 (23.2)	14 (25.0)	1.24 (0.34 - 4.45)	.75
Paresthesia	4 (7.1)	17 (30.4)	··	··
New-onset headache	4 (7.1)	6 (10.7)	··	··
Worsening previous headache	1 (1.8)	10 (17.9)	··	··
Pain	18 (32.1)	17 (30.4)	1.08 (0.51 - 2.29)	.78
Sleep	11 (19.6)	28 (50.0)	9.46 (2.22 - 40.32)	<.001

Abbreviations: OR = Odds ratio.
Data are n (%). Comparison in groups with ≤ 5 are not reported.

a OR presented with 95% CI from logistic regression models with patient id as a random effect. Confidence intervals are based on the Wald statistic
b P-values are based on the likelihood ratio statistic
·· = N/A

© 2023 Peinkhofer C et al. *JAMA Network Open.*

4b Sensitivity analyses tables

eTable 14. Sensitivity Analyses for SCIP Scores in Non-ICU COVID-19 Patients and Non-ICU Hospitalized Controls

	N	Geometric Mean (95% CI)	Estimated Mean (95% CI)	RMD (95%CI)	p value	Het p[a]
Fully adjusted model[b]						
COVID-19	88	58.5 (56.0 - 61.2)	58.2 (55.6 - 61.0)			
AMI	50	61.6 (58.0 - 65.3)	60.9 (57.3 - 64.8)	0.96 (0.89 - 1.03)	.25	
Pneumonia	49	61.8 (58.3 - 65.6)	63.0 (58.9 - 67.3)	0.92 (0.85 - 1.01)	.07	.17
+ adjustment for education years						
COVID-19	88	58.5 (56.2 - 60.9)	58.3 (55.9 - 60.8)			
AMI	50	61.6 (58.4 - 64.9)	61.7 (58.4 - 65.2)	0.94 (0.88 - 1.01)	.11	
Pneumonia	49	61.8 (58.6 - 65.3)	62.1 (58.5 - 66.0)	0.94 (0.87 - 1.01)	.10	.14
+ adjustment for education grade[d]						
COVID-19	88	58.5 (56.1 - 61.0)	58.4 (55.9 - 61.0)			
AMI	50	61.6 (58.2 - 65.1)	61.5 (58.0 - 65.2)	0.95 (0.88 - 1.02)	.17	
Pneumonia	49	61.8 (58.5 - 65.4)	62.2 (58.4 - 66.2)	0.94 (0.87 - 1.02)	.13	.21
+ adjustment for depression						
COVID-19	88	58.5 (56.0 - 61.2)	58.2 (55.6 - 61.0)			
AMI	50	61.6 (58.0 - 65.3)	60.9 (57.3 - 64.8)	0.96 (0.89 - 1.03)	.25	
Pneumonia	49	61.8 (58.3 - 65.6)	63.0 (58.9 - 67.3)	0.93 (0.85 - 1.01)	.07	.17
+ adjustment for alcohol abuse						
COVID-19	88	58.5 (56.0 - 61.2)	58.3 (55.6 - 61.0)			
AMI	50	61.6 (58.0 - 65.3)	60.9 (57.3 - 64.8)	0.96 (0.89 - 1.03)	.25	
Pneumonia	49	61.8 (58.3 - 65.6)	63.0 (58.9 - 67.3)	0.93 (0.85 - 1.01)	.07	.17
+ adjustment for smoking						
COVID-19	88	58.5 (56.0 - 61.2)	57.9 (55.2 - 60.7)			
AMI	50	61.6 (58.0 - 65.3)	61.8 (57.8 - 66.0)	0.94 (0.86 - 1.02)	.13	
Pneumonia	49	61.8 (58.3 - 65.6)	62.8 (58.7 - 67.1)	0.92 (0.85 - 1.00)	.06	.11
+ adjustment for malignancy						
COVID-19	88	58.5 (56.0 - 61.1)	58.1 (55.5 - 60.9)			
AMI	50	61.6 (58.0 - 65.3)	61.2 (57.5 - 65.1)	0.95 (0.88 - 1.03)	.19	
Pneumonia	49	61.8 (58.3 - 65.6)	62.9 (58.9 - 67.2)	0.92 (0.85 - 1.01)	.07	0.14
+ adjustment for previous psychiatric comorbidities						
COVID-19	88	58.5 (56.0 - 61.2)	58.3 (55.6 - 61.0)			
AMI	50	61.6 (58.0 - 65.3)	61.0 (57.3 - 64.8)	0.96 (0.88 - 1.03)	.24	
Pneumonia	49	61.8 (58.3 - 65.6)	62.9 (58.9 - 67.3)	0.93 (0.85 - 1.01)	.08	.17
+ adjustment for previous medical comorbidity						
COVID-19	88	58.5 (56.0 - 61.2)	58.2 (55.5 - 60.9)			
AMI	50	61.6 (58.0 - 65.3)	61.0 (57.4 - 64.9)	0.95 (0.88 - 1.03)	0.22	
Pneumonia	49	61.8 (58.3 - 65.6)	63.0 (59.0 - 67.4)	0.92 (0.85 - 1.00)	.06	.15
+ adjustment for delirium						
COVID-19	88	58.5 (56.0 - 61.2)	58.3 (55.6 - 61.0)			
AMI	50	61.6 (58.0 - 65.3)	60.9 (57.3 - 64.7)	0.96 (0.89 - 1.03)	.26	

© 2023 Peinkhofer C et al. *JAMA Network Open.*

Pneumonia	49	61.8 (58.3 - 65.6)	63.0 (58.9 - 67.3)	0.93 (0.85 - 1.01)	.07	.17
+ adjustment for severity for hospitalization						
COVID-19	88	58.5 (56.0 - 61.2)	58.7 (55.9 - 61.6)			
AMI	50	61.6 (58.0 - 65.3)	60.1 (56.1 - 64.4)	0.98 (0.89 - 1.07)	.59	
Pneumonia	49	61.8 (58.3 - 65.6)	63.0 (59.0 - 67.4)	0.93 (0.85 - 1.01)	.10	.26
Adjusted for all variables						
COVID-19	88	58.5 (56.1 - 61.0)	58.4 (55.6 - 61.2)			
AMI	50	61.6 (58.3 - 65.0)	62.2 (57.9 - 66.8)	0.94 (0.85 - 1.03)	.19	
Pneumonia	49	61.8 (58.5 - 65.3)	61.5 (57.7 - 65.5)	0.95 (0.87 - 1.03)	.21	.29

Abbreviations: AMI = Acute myocardial infarction; Het p = Effect heterogeneity p values; RMD = Relative mean difference; SCIP = Screening for cognitive impairment in psychiatry
Data are presented as number of participants, geometric and estimated means with 95% CI.
a Effect heterogeneity p-values are based on the likelihood ratio statistic
b Fully adjusted models are adjusted for sex, age, admission length and time from hospitalization to follow-up
c Ordinal scale from 1-5 with highest achieved education level: 1, primary school; 2, vocational training or gymnasium; 3, short-cycle higher education; 4, medium-cycle higher education; 5, long-cycle higher education

© 2023 Peinkhofer C et al. *JAMA Network Open.*

**eTable 15.** Sensitivity Analyses for SCIP Scores in ICU COVID-19 and ICU Controls						
	**N**	**Geometric Mean (95% CI)**	**Estimated Mean (95% CI)**	**RMD (95%CI)**	**p value**	**Het p[a]**
Fully adjusted model[b]						
COVID-19	25	59.1 (54.2 - 64.4)	58.7 (53.5 - 64.3)			
ICU	25	60.6 (55.6 - 66.0)	61.0 (55.6 - 66.8)	0.96 (0.84 - 1.10)	.58	.58
+ adjustment for education years						
COVID-19	25	59.1 (54.9 - 63.6)	59.6 (55.1 - 64.5)			
ICU	25	60.6 (56.3 - 65.2)	60.0 (55.4 - 64.9)	0.99 (0.88 - 1.12)	.92	.92
+ adjustment for education grade[d]						
COVID-19	25	59.1 (54.6 - 63.9)	59.9 (54.9 - 65.2)			
ICU	25	60.6 (55.9 - 65.6)	59.8 (54.8 - 65.1)	1.00 (0.88 - 1.14)	.98	.98
+ adjustment for depression						
COVID-19	25	59.1 (54.2 - 64.4)	58.6 (53.4 - 64.3)			
ICU	25	60.6 (55.5 - 66.0)	61.0 (55.7 - 66.9)	0.96 (0.84 - 1.10)	.56	.56
+ adjustment for alcohol abuse						
COVID-19	25	59.1 (54.1 - 64.4)	58.8 (53.5 - 64.6)			
ICU	25	60.6 (55.5 - 66.1)	60.8 (55.4 - 66.9)	0.97 (0.84 - 1.12)	.63	.63
+ adjustment for smoking						
COVID-19	25	59.1 (54.1 - 64.5)	58.2 (52.8 - 64.2)			
ICU	25	60.6 (55.5 - 66.1)	61.4 (55.7 - 67.7)	0.95 (0.81 - 1.10)	.49	.49
+ adjustment for malignancy						
COVID-19	25	59.1 (54.2 - 64.4)	59.0 (53.7 - 64.8)			
ICU	25	60.6 (55.5 - 66.0)	60.6 (55.2 - 66.6)	0.97 (0.84 - 1.12)	.70	.70
+ adjustment for previous psychiatric comorbidities						
COVID-19	25	59.1 (54.3 - 64.3)	58.6 (53.5 - 64.1)			
ICU	25	60.6 (55.6 - 65.9)	61.0 (55.8 - 66.8)	0.96 (0.84 - 1.10)	.55	.55
+ adjustment for previous medical comorbidity						
COVID-19	25	59.1 (54.1 - 64.4)	58.7 (53.5 - 64.4)			
ICU	25	60.6 (55.5 - 66.1)	60.9 (55.5 - 66.8)	0.96 (0.84 - 1.11)	.59	.59
+ adjustment for delirium						
COVID-19	25	59.1 (54.2 - 64.4)	58.9 (53.7 - 64.6)			
ICU	25	60.6 (55.6 - 66.0)	60.7 (55.3 - 66.6)	0.97 (0.84 - 1.12)	.68	.68
+ adjustment for severity for hospitalization						
COVID-19	25	59.1 (54.2 - 64.4)	58.5 (53.3 - 64.2)			
ICU	25	60.6 (55.5 - 66.0)	61.1 (55.7 - 67.1)	0.96 (0.83 - 1.10)	.53	.53
Adjusted for all variables						
COVID-19	25	59.1 (54.4 - 64.1)	59.4 (53.9 - 65.4)			
ICU	25	60.6 (55.8 - 65.7)	60.2 (54.7 - 66.4)	0.99 (0.84 - 1.15)	.85	.85

Abbreviations: Het p = Effect heterogeneity p values; ICU = Intensive care unit; RMD = Relative mean difference; SCIP = Screening for cognitive impairment in psychiatry
Data are presented as number of participants, geometric and estimated means with 95% CI.
a Effect heterogeneity p-values are based on the likelihood ratio statistic
b Fully adjusted models are adjusted for sex, age, admission length and time from hospitalization to follow-up

© 2023 Peinkhofer C et al. *JAMA Network Open.*

c Ordinal scale from 1-5 with highest achieved education level: 1, primary school; 2, vocational training or gymnasium; 3, short-cycle higher education; 4, medium-cycle higher education; 5, long-cycle higher education

© 2023 Peinkhofer C et al. *JAMA Network Open*.

eTable 16. Sensitivity Analyses for MoCA Scores in Non-ICU COVID-19 Patients and Non-ICU Hospitalized Controls

	N	Geometric Mean (95% CI)	Estimated Mean (95% CI)	RMD (95%CI)[a]	p value	Het p[b]
Fully adjusted model[c]						
COVID-19	91	26.5 (25.9 - 27.0)	26.4 (25.8 - 27.0)			
AMI	50	27.2 (26.5 - 27.9)	27.2 (26.4 - 27.9)	0.86 (0.72 - 1.03)	.11	
Pneumonia	50	27.2 (26.4 - 27.8)	27.3 (26.5 - 28.0)	0.85 (0.69 - 1.04)	.11	.15
+ adjustment for education years						
COVID-19	91	26.5 (25.9 - 27.0)	26.4 (25.8 - 27.0)			
AMI	50	27.2 (26.6 - 27.8)	27.3 (26.6 - 27.9)	0.84 (0.70 - 1.00)	.05	
Pneumonia	50	27.2 (26.5 - 27.8)	27.2 (26.4 - 27.8)	0.87 (0.72 - 1.05)	.16	.10
+ adjustment for education grade[d]						
COVID-19	91	26.5 (25.9 - 27.0)	26.4 (25.8 - 27.0)			
AMI	50	27.2 (26.5 - 27.9)	27.3 (26.5 - 27.9)	0.85 (0.71 - 1.02)	.08	
Pneumonia	50	27.2 (26.4 - 27.8)	27.2 (26.4 - 27.9)	0.87 (0.71 - 1.06)	.17	.16
+ adjustment for depression						
COVID-19	91	26.5 (25.9 - 27.0)	26.4 (25.8 - 27.0)			
AMI	50	27.2 (26.5 - 27.9)	27.2 (26.5 - 27.9)	0.86 (0.71 - 1.03)	.10	
Pneumonia	50	27.2 (26.4 - 27.8)	27.3 (26.4 - 28.0)	0.85 (0.69 - 1.04)	.11	.14
+ adjustment for alcohol abuse						
COVID-19	91	26.5 (25.9 - 27.0)	26.4 (25.8 - 27.0)			
AMI	50	27.2 (26.5 - 27.9)	27.2 (26.4 - 27.9)	0.86 (0.72 - 1.03)	.11	
Pneumonia	50	27.2 (26.4 - 27.8)	27.3 (26.5 - 28.0)	0.85 (0.69 - 1.04)	.11	.15
+ adjustment for smoking						
COVID-19	91	26.5 (25.9 - 27.0)	26.4 (25.7 - 27.0)			
AMI	50	27.2 (26.5 - 27.9)	27.3 (26.5 - 28.0)	0.83 (0.68 - 1.02)	.08	
Pneumonia	50	27.2 (26.4 - 27.8)	27.2 (26.4 - 28.0)	0.84 (0.69 - 1.04)	.11	.12
+ adjustment for malignancy						
COVID-19	91	26.5 (25.9 - 27.0)	26.4 (25.8 - 27.0)			
AMI	50	27.2 (26.5 - 27.9)	27.2 (26.5 - 27.9)	0.85 (0.71 - 1.03)	.10	
Pneumonia	50	27.2 (26.4 - 27.8)	27.3 (26.5 - 28.0)	0.85 (0.69 - 1.04)	.11	.14
+ adjustment for previous psychiatric comorbidities						
COVID-19	91	26.5 (25.9 - 27.0)	26.4 (25.8 - 27.0)			
AMI	50	27.2 (26.5 - 27.9)	27.2 (26.5 - 27.9)	0.86 (0.71 - 1.03)	.11	
Pneumonia	50	27.2 (26.4 - 27.8)	27.3 (26.4 - 28.0)	0.85 (0.69 - 1.04)	.12	.15
+ adjustment for previous medical comorbidity						
COVID-19	91	26.5 (25.9 - 27.0)	26.4 (25.7 - 27.0)			
AMI	50	27.2 (26.5 - 27.9)	27.2 (26.5 - 27.9)	0.85 (0.71 - 1.03)	.09	
Pneumonia	50	27.2 (26.4 - 27.8)	27.3 (26.5 - 28.0)	0.84 (0.69 - 1.03)	.10	.12
+ adjustment for delirium						
COVID-19	91	26.5 (25.9 - 27.0)	26.4 (25.8 - 27.0)			
AMI	50	27.2 (26.5 - 27.9)	27.2 (26.4 - 27.9)	0.86 (0.72 - 1.04)	.11	
Pneumonia	50	27.2 (26.4 - 27.8)	27.3 (26.5 - 28.0)	0.85 (0.69 - 1.04)	.11	.15

© 2023 Peinkhofer C et al. *JAMA Network Open.*

+ adjustment for severity for hospitalization						
COVID-19	91	26.5 (25.9 - 27.0)	26.6 (25.9 - 27.2)			
AMI	50	27.2 (26.5 - 27.9)	26.9 (26.0 - 27.7)	0.94 (0.76 - 1.17)	.59	
Pneumonia	50	27.2 (26.4 - 27.8)	27.3 (26.5 - 28.0)	0.87 (0.71 - 1.08)	.20	.44
Adjusted for all variables						
COVID-19	91	26.5 (25.9 - 27.0)	26.5 (25.9 - 27.1)			
AMI	50	27.2 (26.5 - 27.8)	27.2 (26.3 - 28.0)	0.88 (0.69 - 1.11)	.27	
Pneumonia	50	27.2 (26.4 - 27.8)	27.1 (26.2 - 27.8)	0.90 (0.74 - 1.11)	.32	.44

Abbreviations: AMI = Acute myocardial infarction; Het p = Effect heterogeneity p values ; MoCA= Montreal cognitive assessment¸ RMD = Relative mean difference
Data are presented as number of participants, geometric and estimated means with 95% CI.
a Relative mean difference for MoCA scores refers to mean difference of “32 – MoCA score”
b Effect heterogeneity p-values are based on the likelihood ratio statistic
c Fully adjusted models are adjusted for sex, age, admission length and time from hospitalization to follow-up
d Ordinal scale from 1-5 with highest achieved education level: 1, primary school; 2, vocational training or gymnasium; 3, short-cycle higher education; 4, medium-cycle higher education; 5, long-cycle higher education

© 2023 Peinkhofer C et al. *JAMA Network Open.*

eTable 17. Sensitivity Analyses for MoCA Scores in ICU COVID-19 and ICU Controls

	N	Geometric Mean (95% CI)	Estimated Mean (95% CI)	RMD (95%CI)[a]	p value	Het p[b]
Fully adjusted model[c]						
COVID-19	29	26.4 (25.2 - 27.4)	26.5 (25.3 - 27.5)			
ICU	25	27.7 (26.7 - 28.5)	27.5 (26.5 - 28.4)	0.81 (0.60 - 1.11)	.19	.19
+ adjustment for education years						
COVID-19	29	26.4 (25.4 - 27.2)	26.7 (25.7 - 27.6)			
ICU	25	27.7 (26.8 - 28.4)	27.4 (26.4 - 28.2)	0.87 (0.66 - 1.15)	.33	.33
+ adjustment for education grade[d]						
COVID-19	29	26.4 (25.4 - 27.2)	26.8 (25.8 - 27.6)			
ICU	25	27.7 (26.8 - 28.4)	27.3 (26.3 - 28.1)	0.91 (0.69 - 1.21)	.52	.52
+ adjustment for depression						
COVID-19	29	26.4 (25.2 - 27.4)	26.5 (25.3 - 27.5)			
ICU	25	27.7 (26.7 - 28.5)	27.5 (26.4 - 28.4)	0.81 (0.59 - 1.12)	.19	.19
+ adjustment for alcohol abuse						
COVID-19	29	26.4 (25.2 - 27.4)	26.5 (25.2 - 27.5)			
ICU	25	27.7 (26.7 - 28.5)	27.6 (26.5 - 28.5)	0.80 (0.58 - 1.11)	.18	.18
+ adjustment for smoking						
COVID-19	29	26.4 (25.2 - 27.4)	26.5 (25.1 - 27.5)			
ICU	25	27.7 (26.7 - 28.5)	27.6 (26.4 - 28.5)	0.79 (0.56 - 1.12)	.18	.18
+ adjustment for malignancy						
COVID-19	29	26.4 (25.2 - 27.4)	26.5 (25.2 - 27.5)			
ICU	25	27.7 (26.7 - 28.5)	27.6 (26.5 - 28.5)	0.80 (0.58 - 1.11)	.18	.18
+ adjustment for previous psychiatric comorbidities						
COVID-19	29	26.4 (25.2 - 27.3)	26.6 (25.4 - 27.5)			
ICU	25	27.7 (26.7 - 28.5)	27.5 (26.4 - 28.4)	0.83 (0.61 - 1.12)	.22	.22
+ adjustment for previous medical comorbidity						
COVID-19	29	26.4 (25.2 - 27.4)	26.5 (25.3 - 27.5)			
ICU	25	27.7 (26.7 - 28.5)	27.5 (26.4 - 28.4)	0.82 (0.59 - 1.12)	.20	.20
+ adjustment for delirium						
COVID-19	29	26.4 (25.2 - 27.4)	26.5 (25.3 - 27.5)			
ICU	25	27.7 (26.7 - 28.5)	27.5 (26.4 - 28.4)	0.82 (0.60 - 1.12)	.21	0.21
+ adjustment for severity for hospitalization						
COVID-19	29	26.4 (25.2 - 27.3)	26.5 (25.3 - 27.5)			
ICU	25	27.7 (26.7 - 28.5)	27.6 (26.5 - 28.5)	0.80 (0.58 - 1.09)	.15	.15
Adjusted for all variables						
COVID-19	29	26.4 (25.4 - 27.2)	26.4 (25.2 - 27.4)			
ICU	25	27.7 (26.8 - 28.4)	27.6 (26.6 - 28.5)	0.78 (0.57 - 1.07)	.12	.12

Abbreviations: Het p = Effect heterogeneity p values; ICU = Intensive care unit; MoCA= Montreal cognitive assessment; RMD = Relative mean difference
Data are presented as number of participants, geometric and estimated means with 95% CI.
a Relative mean difference for MoCA scores refers to mean difference of “32 – MoCA score”
b Effect heterogeneity p-values are based on the likelihood ratio statistic
c Fully adjusted models are adjusted for sex, age, admission length and time from hospitalization to follow-up

© 2023 Peinkhofer C et al. *JAMA Network Open.*

d Ordinal scale from 1-5 with highest achieved education level: 1, primary school; 2, vocational training or gymnasium; 3, short-cycle higher education; 4, medium-cycle higher education; 5, long-cycle higher education

© 2023 Peinkhofer C et al. *JAMA Network Open*.

eFigure 1. Detailed Flowchart of Inclusion Process

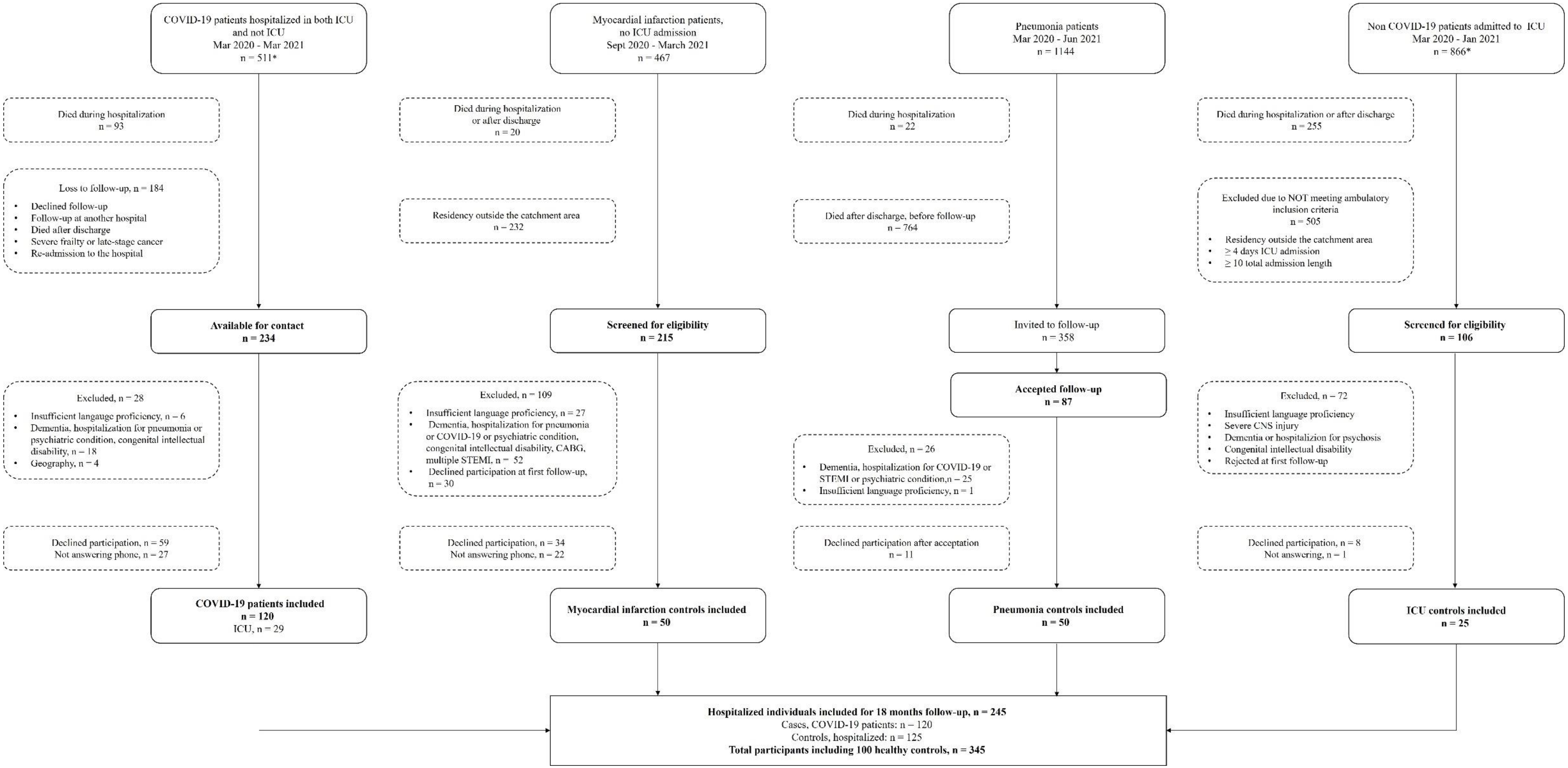

*56 (11%) COVID-19 subjects were hospitalized at another hospital at the same time-period and considered for follow-up; 3 ICU patients were included from the pneumonia group.

© 2023 Peinkhofer C et al. *JAMA Network Open.*

eFigure 2. Secondary Outcomes Compared Between COVID-19 and Control Groups

A-C: Relative mean difference secondary cognitive and psychiatric outcomes. The arrow at the bottom of each subfigure signifies whether there is an improvement or a worsening in the corresponding score within the COVID-19 group when compared to the control groups. The right-pointing arrow indicates that a higher score corresponds to a worse performance. For example, in Trail A and B, a higher score denotes more time taken to complete the test, thereby indicating a poorer performance**.**

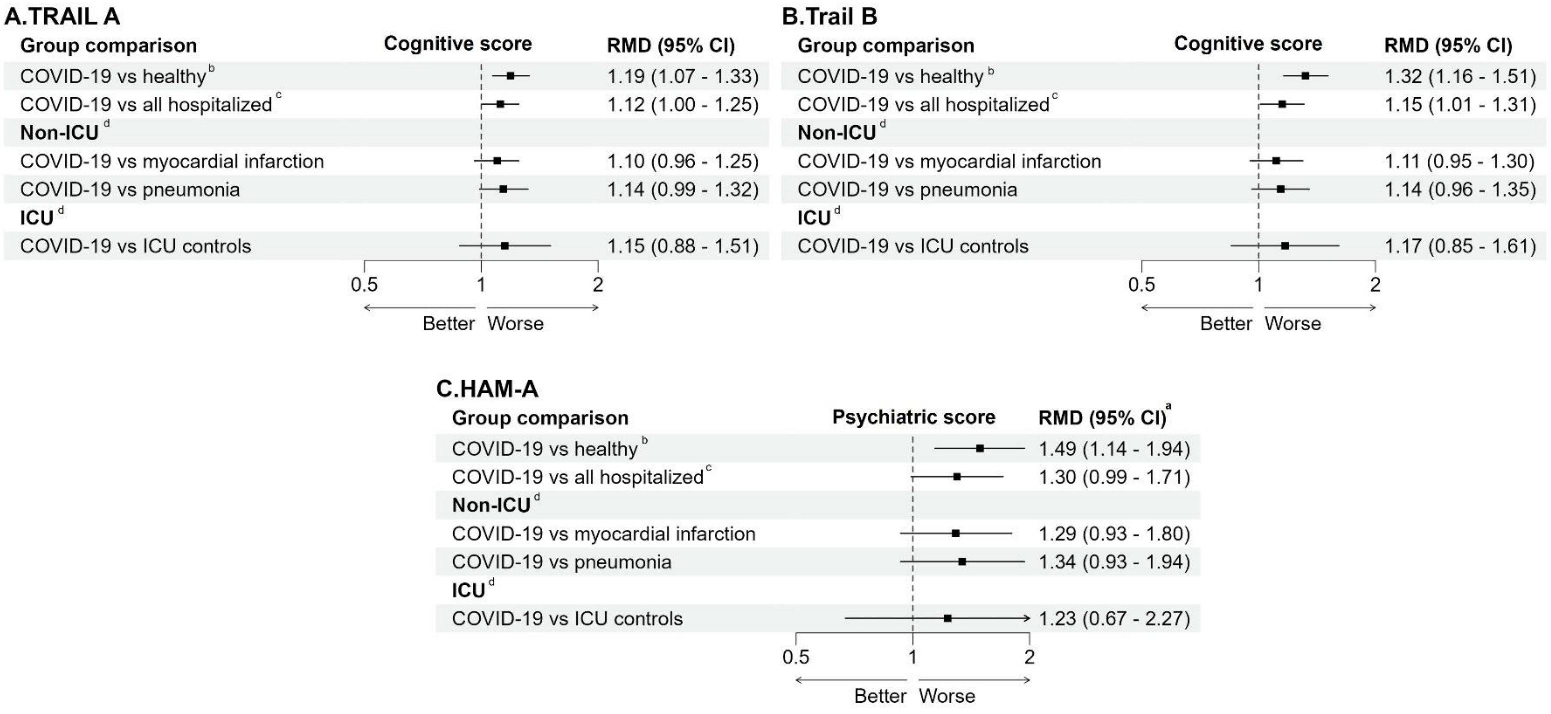

[a] Relative mean difference for Hamilton Anxiety scores refers to "total score +1" ; [b] Models are adjusted for sex, age, BMI, education grade, alcohol abuse, smoking and ICU admission; [c] Models are adjusted for sex, age, admission length and time from hospitalization to follow-up, ICU admission and severity; [d] Models are adjusted for sex, age, admission length and time from hospitalization to follow-up. Abbreviations: HAM-A = Hamilton Anxiety; ICU = Intensive care unit; RMD = Relative mean difference; SCIP = Screening for Cognitive Impairment in Psychiatry.

© 2023 Peinkhofer C et al. *JAMA Network Open.*

eFigure 3. MoCA Scores in COVID-19 Cases and Controls at 18-Month Follow-up and Changes in Mean MoCA Scores Over Time in COVID-19 Cases

A. Mean MoCA scores in models adjusted for sex, age, BMI, education grade, alcohol abuse, smoking and ICU admission (COVID-19 versus healthy controls) and sex, age, admission length, time from hospitalization to follow-up, ICU admission and severity (COVID-19 versus hospitalized individuals). Error bars indicate 95% CI. Only significant p values are reported. **B.** Mean MoCA scores in linear models with patient id as a random effect. MoCA scores were available for 16 COVID-19 patients at discharge, 56 at 6 months and 120 at 18 months follow-up. Error bars indicate 95% CI. Only significant p values are reported. * p ≤.05, ** p ≤.01; ***p≤.001.

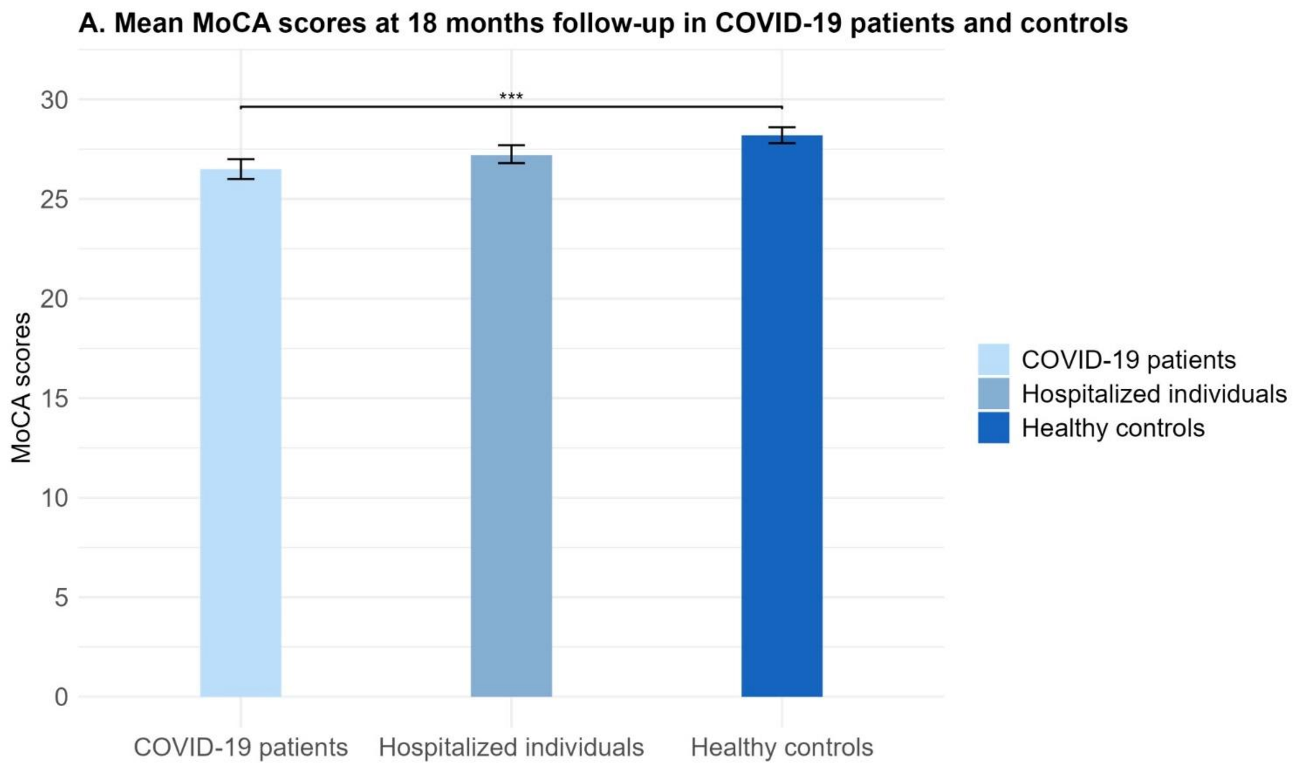

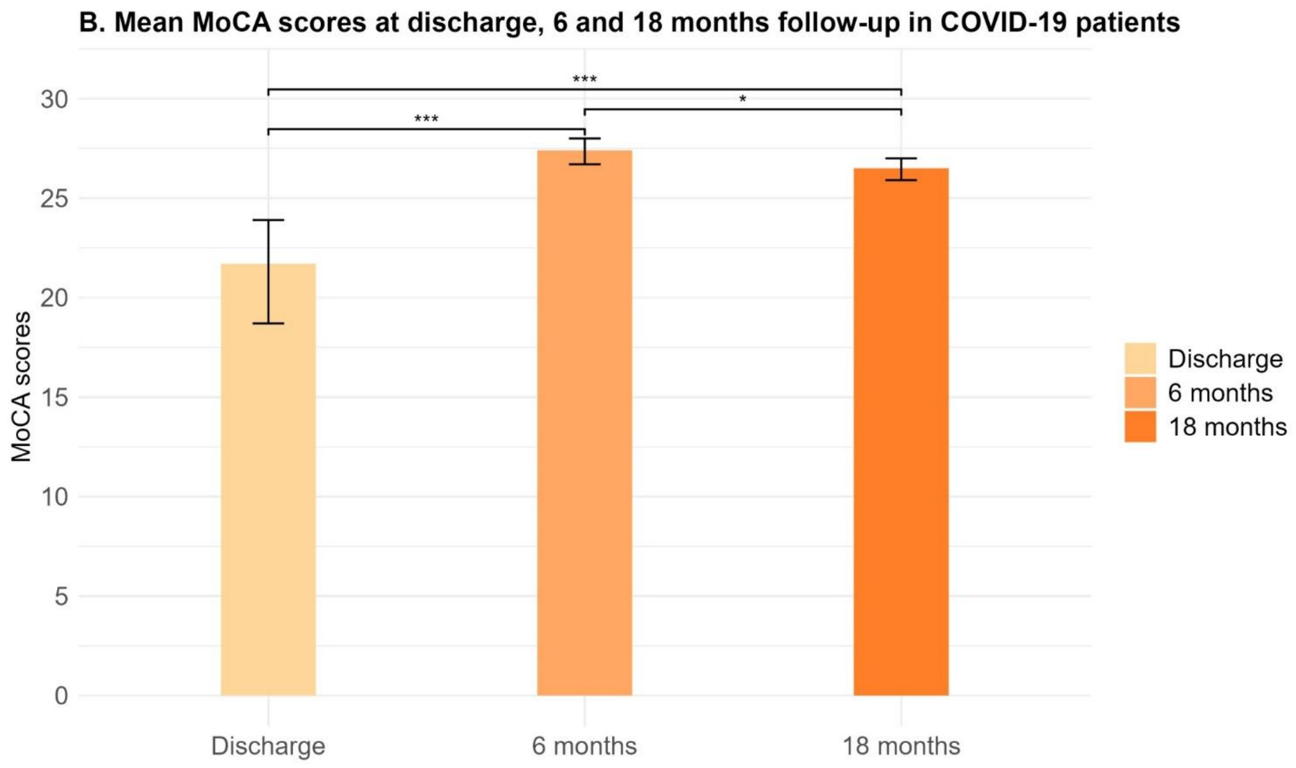

© 2023 Peinkhofer C et al. *JAMA Network Open*.

eFigure 4. Radar Chart Showing Trajectories of Psychiatric Symptoms and Diagnoses (A) and Neurological Symptoms and Signs (B) From 6 to 18 Months After COVID-19 Hospitalization

Frequency of findings at the two time points was available for 56 COVID-19 patients. Results are based on logistic regression models with patient id as a random effect. * p ≤.05

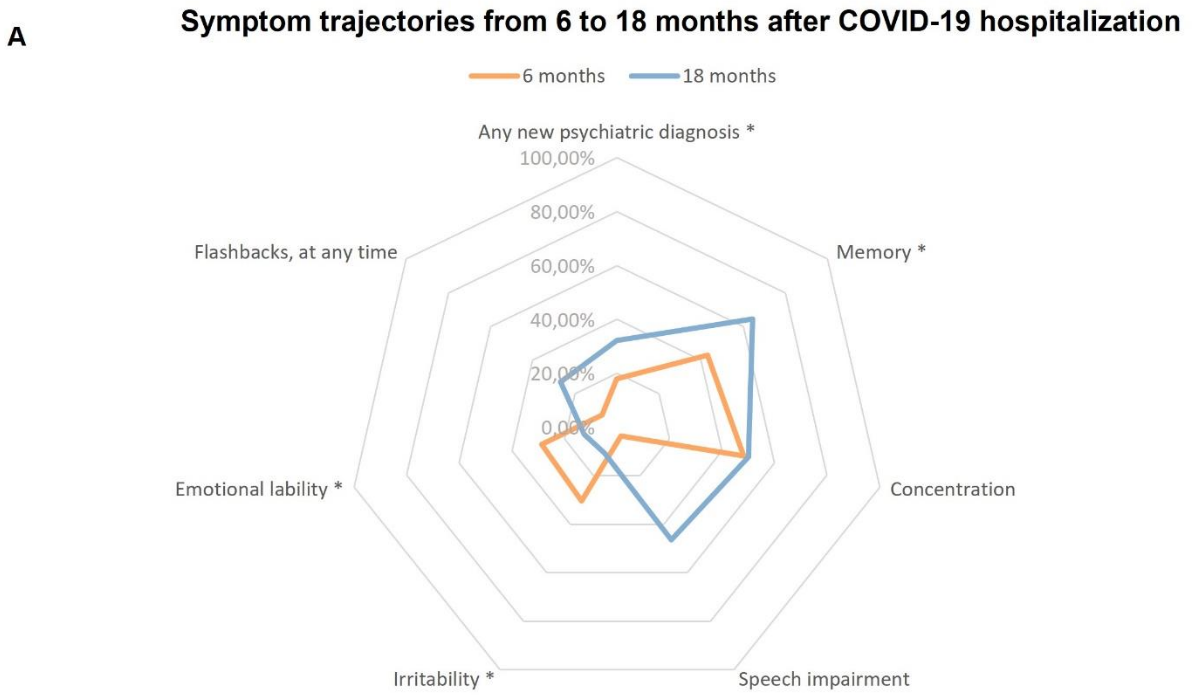

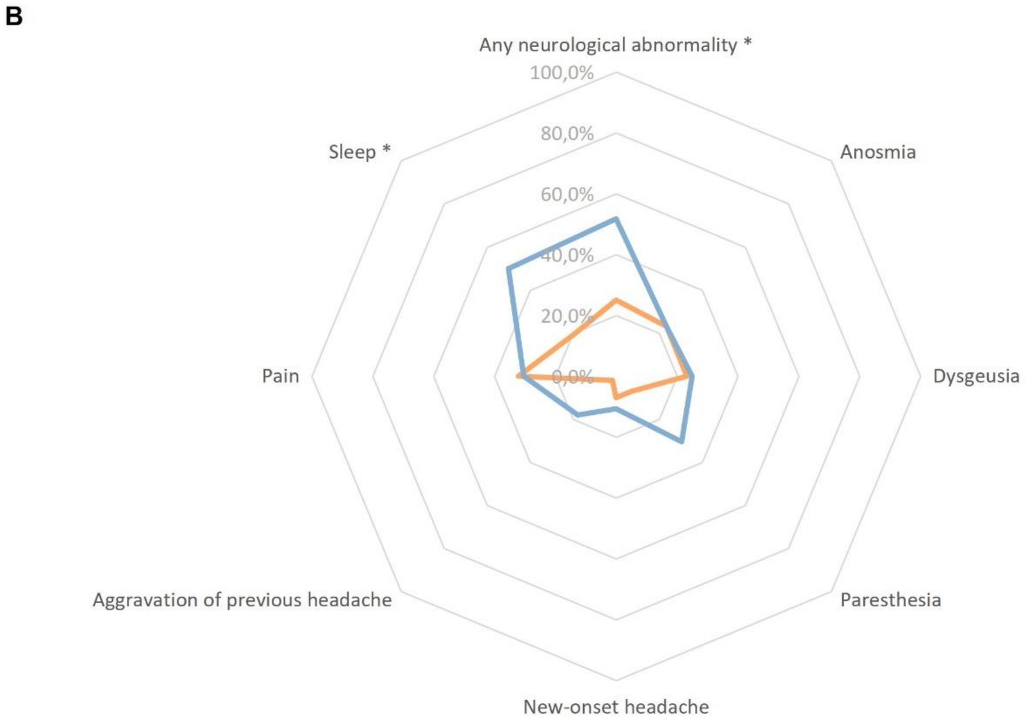

© 2023 Peinkhofer C et al. *JAMA Network Open.*

eReferences.

1 Nersesjan V, Amiri M, Lebech AM, *et al.* Central and peripheral nervous system complications of COVID-19: a prospective tertiary center cohort with 3-month follow-up. *J Neurol* 2021; **268**: 3086–104.

2 Nersesjan V, Fonsmark L, Christensen RHB, *et al.* Neuropsychiatric and Cognitive Outcomes in Patients 6 Months after COVID-19 Requiring Hospitalization Compared with Matched Control Patients Hospitalized for Non-COVID-19 Illness. *JAMA Psychiatry* 2022; **79**. DOI:10.1001/jamapsychiatry.2022.0284.

© 2023 Peinkhofer C et al. *JAMA Network Open.*
