# Supplementary material for: Brain Health After COVID-19, Pneumonia, Myocardial Infarction, or Critical Illness
Source: JAMA Netw Open. 2023 Dec 28;6(12):e2349659. doi: 10.1001/jamanetworkopen.2023.49659 (PMC10755623; doi:10.1001/jamanetworkopen.2023.49659)
Supplement: Supplement 2. — Data Sharing Statement [file jamanetwopen-e2349659-s002.pdf]

Data Sharing Statement

Peinkhofer. Brain Health After COVID-19, Pneumonia, Myocardial Infarction, or Critical Illness. *JAMA Netw Open*. Published December 28, 2023. doi:10.1001/jamanetworkopen.2023.49659

Data
Data available: Yes
Data types: Deidentified participant data
How to access data: daniel.kondziella@regionh.dk
When available: With publication

Supporting Documents
Document types: None

Additional Information
Who can access the data: Anyone with reasonable request
Types of analyses: for replication
Mechanisms of data availability: investigator support
